# NPCoronaPredict: A Computational Pipeline for the Prediction of the Nanoparticle–Biomolecule Corona

**DOI:** 10.1021/acs.jcim.4c00434

**Published:** 2024-09-26

**Authors:** Ian Rouse, David Power, Julia Subbotina, Vladimir Lobaskin

**Affiliations:** University College Dublin, Belfield, Dublin 4, Ireland

## Abstract

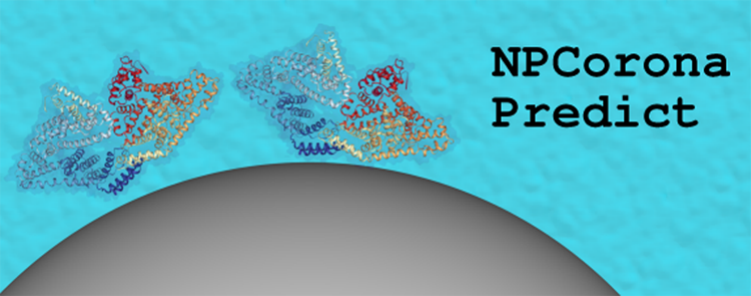

The corona of a nanoparticle immersed in a biological fluid is of key importance to its eventual fate and bioactivity in the environment or inside live tissues. It is critical to have insight into both the underlying bionano interactions and the corona composition to ensure biocompatibility of novel engineered nanomaterials. A prediction of these properties in silico requires the successful spanning of multiple orders of magnitude of both time and physical dimensions to produce results in a reasonable amount of time, necessitating the development of a multiscale modeling approach. Here, we present the NPCoronaPredict open-source software package: a suite of software tools to enable this prediction for complex multicomponent nanomaterials in essentially arbitrary biological fluids, or more generally any medium containing organic molecules. The package integrates several recent physics-based computational models and a library of both physics-based and data-driven parametrizations for nanomaterials and organic molecules. We describe the underlying theoretical background and the package functionality from the design of multicomponent NPs through to the evaluation of the corona.

## Introduction

Advanced materials represent a new paradigm in materials science: substances with highly specific features and enhanced target properties derived from precise control over their structure and composition. A particularly relevant set of examples of these materials are nanomaterials, which may exhibit properties that significantly differ from the expected behavior of the same bulk material due to the high surface-to-volume ratio. The large surface implies high specific reactivity and capacity for steering complex processes at the molecular level. New materials, however, come with new risks: these same desirable properties may also lead to unwanted behavior when these novel materials come into contact with the environment or living beings.^1,2^ As with the benefits, these risks are high for nanomaterials, since their small size enables rapid uptake by the body through multiple pathways, e.g., inhalation, ingestion, or skin contact. Consequently, it is important to be able to predict whether a given material is toxic or biocompatible early at the stage of the material’s development.^3^ Given the vast range of materials used in modern technology or considered as candidates for applications, and in light of the general need to reduce the amount of in vivo and in vitro tests performed, this suggests the use of in silico methods to predict bioactivity from first principles.^4^

To date, the main focus of experimental studies—and thus the initial goal for computational methods designed to predict experiments—for the bioactivity of nanoparticles (NP) has been focused on the protein corona: the layer of proteins directly and strongly bound to the surface, the hard corona, of the NP and the soft corona of molecules adsorbed to these inner proteins.^5,6^ Recently, growing attention has also been paid to the fact that the corona need not consist only of proteins.^7^ Other molecules, be they metabolites, peptide fragments, lipids, or small organic molecules such as hormones, medicine, or toxins will also adsorb to the NP and likewise be transported along with it, and these may completely alter the final destination and biological outcomes, whether this is deliberate (as in a drug nanocarrier) or accidental. Thus, the computational methodology relating to the corona should be sufficiently general to account for a wide variety of biomolecules, or indeed arbitrary organic molecules, in addition to proteins. The relative binding affinity of these constituents and, hence, the corona composition is controlled by the molecular-level properties of the NP surface: the type of atoms and their connectivity, their partial charge, polarizability, density, and larger-scale geometrical features such as the crystal structure and its curvature. To be able to connect these properties to the corona composition, these must all be factored into the computational methodology for corona prediction. Moreover, in the context of advanced materials, the methodology must allow for the combination of various structural elements (core, shell, dopants, functional groups) defined in terms of their specific properties into a composite NP that reflects its structural complexity and relates it to the characteristics of the corona.

A key challenge on this path is bridging the length- and time scale gaps between the fundamental features of the materials and the high-level properties of interest, which can amount to several orders of magnitude.^4^ On the one hand, the adsorption of a single molecule to an NP is highly dependent on the local atomic structure and presence of solvent, and thus this requires the use of atomistic-level methods. The quantum and classical atomistic methods, however, require enormous resources to scale up to the adsorption of a single protein for the time scale of milliseconds. On the other hand, a typical corona may consist of hundreds of adsorbates and develops over the course of hours. Thus, it would be prohibitively expensive to simulate the corona for a single NP in a single medium, let alone scan over multiple NPs or biological environments using a brute-force atomistic simulation. To overcome this, it is necessary to employ coarse-graining methods to allow for longer time scales and larger systems to be reached while maintaining physical accuracy and connection to the original material. Given the wide range of potential adsorbates and NPs, these methods must be sufficiently generic to cover as many possibilities as possible while remaining accessible enough such that a novice user can perform corona predictions without extensive training. A key advantage is granted by the fact that the vast majority of biomolecules, and proteins in particular, can be represented using a relatively small number of simple repeat units such as amino acids (AA) or sugars. It is reasonable to precalculate the interaction for these building blocks and use these to construct a model for the entire biomolecule, or family of related biomolecules, thus greatly reducing the amount of effort that must be expended to evaluate the total adsorption energy or parametrize a new biomolecule. Likewise, although NPs can in principle be highly complex, they too can be subdivided into interchangeable components such as solid or hollow spheres or cylinders of simple materials, and these can be used to construct multicomponent NP step-by-step. This has led us to the development of a series of increasingly complex models for protein adsorption, starting from an initial simple model using Lennard-Jones (LJ)-like interactions between NPs and AAs,^8^ to a more complex model of protein adsorption to gold^9^ or titanium dioxide^10^ and more recently including multiple NP components simultaneously.^11^ We have further developed models for the prediction of the corona, taking advantage of binding energies computed using the protein–NP models and allowing for a simulation of competitive adsorption in media with a large number of possible adsorbates.^12−14^

**Figure 1:**
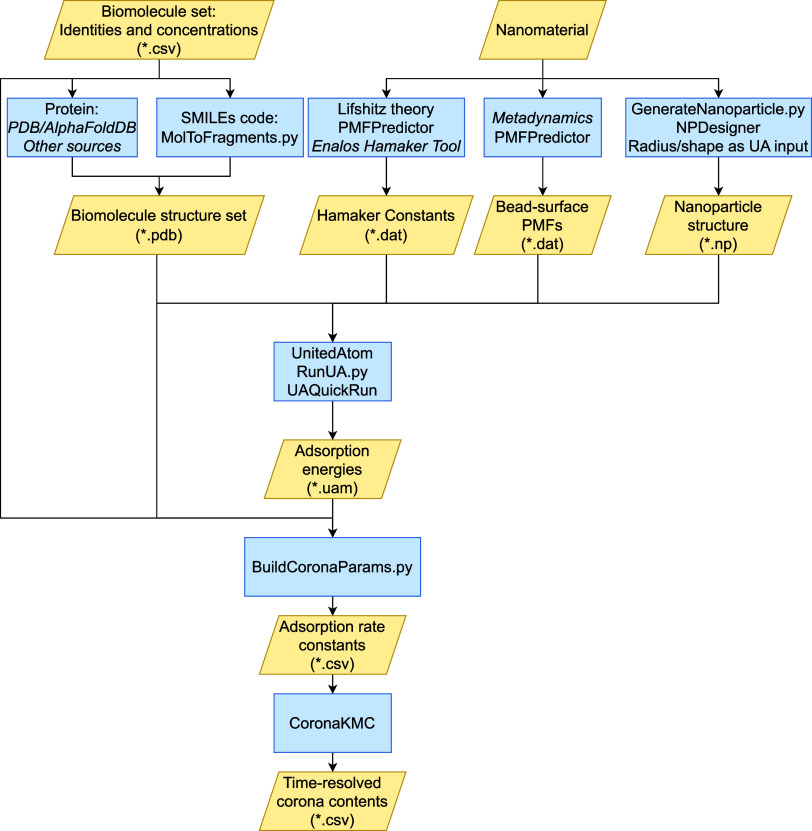
A summary of the overall workflow for corona prediction using the suite of computational tools discussed here. Yellow parallelograms indicate data used as input/output with the expected file format indicated in brackets. Blue rectangles indicate software or methodologies used to generate or process input, with entries in italics indicating external methodologies not included in either NPCoronaPredict or PMFPredictor. A large set of precomputed Hamaker constants and Bead-surface PMFs are additionally supplied in the repository for immediate use.

In this work, we present a description of NPCoronaPredict, the computational pipeline we have developed to enable the prediction of the corona for NPs immersed in solution containing multiple potential adsorbates—typically, but not necessarily, of biological origin—with an overview of the generic workflow presented in Figure 1. The basis of this material-specific prediction is a set of interaction potentials based on the atomistic structure of the NP surface for an example of each material and the small molecules or molecular fragments of interest, taking into account the presence of solvent and ions as necessary. Correction functions are applied by the software to these potentials to convert from the example geometry to the actual geometry for the NP of interest and used as building blocks to construct potentials for more complex macromolecules. This reuse of potentials substantially reduces the required computational time which would otherwise be required while still preserving details from the initial atomistic simulations. The required input potentials are supplied for a range of materials obtained via atomistic simulations and cover the adsorption of AA side chain analogues (SCAs), lipid fragments and sugars to a range of carbonaceous, metallic, and metal oxide structures.^9−11,15−17^

In brief, the methodology proceeds as follows. The user specifies a target NP or complex of nanoparticles NPs, selecting from a list of available materials and assigning a shape and radius as desired, and provides a list of molecules and concentrations present in the medium. Each macromolecule, e.g., a protein, is decomposed into a set of coarse-grained beads, e.g., individual AAs. The geometry-corrected interactions between each bead type and the nanomaterial surface are computed and used to create an overall interaction potential between the NP and macromolecule by summation over all beads. Adsorption energies at a range of relative orientations are extracted from this potential and used to estimate adsorption and desorption rate constants for each orientation of each molecule. These are used as input to a kinetic Monte Carlo (KMC) simulation of the corona formation, which provides numbers of each type of adsorbate present in the corona as a function of time.

We stress that the general methodology is by no means limited to these surfaces or biomolecular fragments, or indeed to considering only proteins or other biomolecules. To take advantage of this, the repository also contains a large databank of input potentials generated for a wider range of surfaces and approximately 200 small organic molecules generated via a machine-learning (ML) method (PMFPredictor) based on atomistic force fields for the materials and molecules.^18,19^ This approach enables the rapid generation of even further input potentials for surfaces given an atomistic force field and structure, while new small molecules can be generated and parametrized using the GAFF force field^20^ and acpype^21^ software via their SMILES code. More generally, the user is free to parametrize their own surfaces or chemicals as required to extend the NPCoronaPredict software suite to their own particular needs through their own preferred methodology, and the software is designed to be agnostic to the source of these inputs, although we recommend the use of the PMFPredictor methodology since this is designed to produce output compatible with NPCoronaPredict and is available open-source.^19^ We further extend the functionality by providing software tools for decomposing larger organic molecules, e.g., drug candidates, into fragment-based models compatible with this software.

Our multiscale approach has been developed to take advantage of the UnitedAtom and CoronaKMC methodologies first described elsewhere^9,12^ while expanding these to cover a far greater range of use cases beyond the adsorption of proteins first considered, and provides a convenient pipeline to enable prediction of the corona with minimal user intervention. In particular, the corona for a wide range of simple NPs consisting of a single material type and fixed radius can be generated for a target mixture of proteins and other biomolecules by running a single command or via a graphical interface. We have also significantly improved the ability of the software to handle complex cases such as proteins with concave or hollow regions into which a small NP may dock. The repository as described in this work can be obtained via git at^22^ and corresponds to Release v1.0.4. The previous version of the code stored at the former UnitedAtom package location^23^ is outdated and should no longer be used, but is kept for historical purposes. Since the code remains in active development future versions may have altered behavior compared to the version described here, but this will be indicated via updated version numbers. A C++ compiler with the boost libraries and headers installed is required to compile UnitedAtom, while CoronaKMC requires a Python 3 installation with full dependencies given in the documentation. A QT installation is also required to compile optional graphical interfaces. In the following sections, we provide a detailed description of each component of the suite, including the underlying methodology, required inputs and expected outputs as well as examples of usage and validation of individual components.

## Package Overview

The NPCoronaPredict package consists of two main components, UnitedAtom and CoronaKMC to predict biomolecule–NP adsorption energies and corona contents respectively via a multiscale coarse-grained approach. It also contains a number of additional scripts and tools to simplify the use of these modules, prepare input, and enable a comprehensive characterization of the corona formed by a particular NP in a given medium.

These scripts are interconnected as shown in Figure 1, which provides an overview of the tools included and the data required as input for each step. The supplied NPCoronaPredict.py script and NPCoronaPredict-GUI tool automate many of these steps, requiring only that the user provide a list of biomolecules of interest and choose a material from the predefined options provided in the Material Library, then select a geometry and size for the NP. All remaining steps are then performed automatically by the software. However, the general procedure remains the same if the user chooses to run each component manually. First, UnitedAtom computes binding energies for each target biomolecule by summing over interaction potentials between small fragments of the biomolecule and the NP. This requires the Hamaker constants and Bead-surface PMFs noted in Figure 1 as input, which we provide for a wide range of template materials and which are automatically adapted to the particular geometry of the NP requested by the user by the software. The resulting adsorption energies are then translated to rate constants for adsorption and desorption for each orientation and output to a new file by a Python script supplied in the repository. The second main program, CoronaKMC, reads this file as input and performs a KMC simulation of the entire corona, outputting the number of each type of biomolecule adsorbed to the surface of the NP as a function of time.

## UnitedAtom: Biomolecule–Nanoparticle Adsorption Affinity

The UnitedAtom (hereafter UA and stylized as a single word to distinguish from generic united atom methodologies) software tool is designed to efficiently calculate the adsorption energy of rigid biomolecules consisting of well-defined repeat units to an NP through a coarse-grained (CG) methodology. Throughout, we use “biomolecule” to refer to a rigid structure consisting of one or more CG adsorbate beads (ABs). The NP is represented in terms of one or more units we refer to as NP beads (NPBs), which represent a simple geometrical shape (sphere/cylinder/cube) of a specified material. Thus, the NP itself is also coarse-grained. The general methodology has been published in detail elsewhere^9−11^ and is summarized in this work. In overview, for a specific simulation configuration and input set consisting of a biomolecule structure and an NP, the pairwise interaction potentials between each NPB and AB is used to produce an overall interaction potential between the biomolecule in a specific orientation and the NP as a function of the distance between the centers of mass of the two objects. From this potential, an orientation-specific adsorption energy is extracted and recorded. This procedure is repeated for the full range of orientations of the biomolecule relative to the NP to produce a table of adsorption energies. In the following sections, we provide an overview of the methodology and discuss the required inputs in more detail.

### Methodology

When executed, the UA program performs the following steps: construction of potentials for each type of AB component of the large biomolecule to each type of NPB, generation of samples of different orientations of the biomolecule relative to the NP, summation of the interaction potentials over all the AB as a function of their position in the biomolecule–NP complex, and integration of the resulting total potential over distance to produce the adsorption energy. The required potentials are primarily generated from precomputed, material-specific sets of tabulated potentials for each AB, usually PMFs computed through atomistic simulations. Although these must be externally computed for each nanomaterial, this need only be done for one geometry per material, e.g., a planar slab, since UA applies corrections to remap these tabulated potentials to spheres, cylinders or cubes of arbitrary size. The remaining potentials are computed on-the-fly by UnitedAtom from known expressions for the Hamaker and electrostatic potentials, requiring only Hamaker constants and surface electrostatic potentials.

First, the NP structure is generated or loaded. A bounding radius *R*_0_^b^, representing the solid core of the NP is computed from the outer radius of the largest NPB if this is not manually set in the configuration file. An outermost bounding radius *R*_1_^b^ is computed from max(|*r_n_*| + *R_n_*), where |*r_n_|* is the distance of the bead center from the origin and *R_n_* is the radius of the NPB, if this value has not been manually set. This methodology is chosen to produce reasonable results for an NP consisting of a core and brush configuration, for which the inner radius encapsulates the core and the outer radius ensures that all of the brush is included. We note that these automatically assigned values can be manually overridden if desired, which may be necessary for NPs without a well-defined core such as agglomerates. Next, the required interaction parameters for all target AB types are loaded and the interaction potentials between each type of NPB and AB are computed. We discuss these in more detail later, but in brief these potentials include a short-range tabulated potential which is typically a potential of mean force (PMF), and long-range Hamaker and electrostatic components. In the default methodology, the interaction between an AB type (ALA, GLY, etc.) indexed *m* and the total NP complex is then computed along a single axis by summation over all NPBs indexed *n*, that is
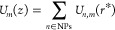
1where r* = r*(*x*_*n*_, *y*_*n*_, *z*_*n*_, *z*, *R*_*n*_) is the geometry-specific closest approach of a AB center at (0, 0, *z*) to the NPB of size *R*_*n*_ and with center at (*x*_*n*_, *y*_*n*_, *z*_*n*_), and the input potentials depend on the particular AB–NPB pair and are described later. If this presummation is disabled, then the computed potentials for each NPB and AB type *U*_*n*,*m*_ are instead stored in memory for later use.

With the NP structure defined, the biomolecules of interest are loaded sequentially and orientational sampling is performed. First, the biomolecule is shifted such that its center of mass (COM) is defined to be (0, 0, 0) and rotations are applied to set the biomolecule to the target orientation. This orientation is defined by two angles ϕ, θ and optionally a third angle ω if provided. The input structure for a given biomolecule is rotated by an angle equal to −ϕ around the z-axis, followed by a rotation of 180°−θ degrees around the *y*-axis. Depending on the selected geometry and configuration options, a final rotation of ω around the z-axis may then be applied; this functionality is disabled for basic spheres by default but can be manually enabled for anisotropic NPs and is automatically enabled for cylindrical NPs. Following this rotation, the vector originally defined by (cos ϕ sin θ, sin ϕ sin θ, cos θ) is mapped to (0, 0, −1), which is normal to and pointing toward the surface of the NP, while the angle ω produces a rotation around this axis or, equivalently, a rotation of the NP. Note that if the NP is symmetric with respect to rotation around the z-axis the rotation around ω will not change the final output. We further note that in the original frame of reference of the biomolecule as specified in the input .pdb file, the NP is located at spherical coordinates given by ϕ, θ. The biomolecule is then translated along the line (0, 0, *z*) to define its location at a fixed NP–offset distance *h*, where a range of values of *h* are sampled during the calculation according to limits discussed later.

In the default presummation model, the NP complex–biomolecule potential is then obtained by summation of *U*_*m*_(*z*) over all ABs indexed *i*,
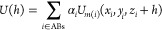
2where *x*_*i*_, *y*_*i*_, *z*_*i*_ are the bead locations defined by the geometry of the molecule and *h*, ϕ, θ, ω, the AB type for bead *i* is denoted *m*(*i*), and α_*i*_ is a per-residue weight, with *x*_*i*_, *y*_*i*_, *z*_*i*_, α_*i*_ read from the input file as discussed in Section “ Biomolecule Definition”. This default behavior performs acceptably well for isotropic NPs but does not produce meaningful results for an NP decorated with a brush, for which the potential experienced by a given AB depends on all coordinates and not just its distance from the NP. In this case, we recommend disabling presummation such that the potential is instead given by
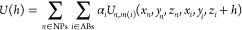
3This double summation substantially increases the required computational time but produces a more physically accurate result. If presummation is not disabled, the physical geometry of the NP and presence of brush beads is accounted for in a less accurate way by imposing a small “overlap penalty” if any AB is determined to be in a location which would overlap with one of the NPBs. For reasons of numerical stability, an additional extreme-short-range potential is also applied in both of these summation models to prevent the calculation diverging when sampling reasons of space in which an NP and AB overlap, which we define to be when the center of the AB is at a distance of under 0.1 nm from the surface of the NPB. This potential is not directly parametrizable by the user and has the form *U*_*x*_(*h*) = (0.1/*h*)^12^ – 1.0 in units *k*_B_*T* and is set to 0 for *h* > 0.1 nm such that it does not contribute to the potential in realistic conformations.

Once the potential summed over all ABs is obtained, UA then performs a free-energy integration,
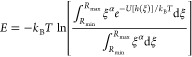
4where α = 2 for spherical coordinates, α = 1 for cylindrical coordinates and α = 0 for planar systems, and we use the variable ξ to represent the distance from the center of the NP to the COM of the biomolecule. By default, *T* = 300 K but this parameter may be set in the configuration file. The bounds of integration are automatically chosen based on the NP geometry and the structure of the biomolecule as follows. The inner bound *R*_min_ gives the closest approach of the COM of the biomolecule to the COM of the NP complex and is computed from the structure of the biomolecule and the NP binding radius *R*_0_^b^ using one of two methodologies. The first, applied by default, is chosen to approximate the situation of the biomolecule approaching from infinity along the *z*-axis and stopping at first contact with the NP, i.e., at the maximum COM–COM distance such that a bead of the biomolecule is in contact with the NP or when the COM–COM distance is equal to zero, whichever occurs first. The second, chosen if the user enables the “full-scan” mode, instead chooses this lower bound such that the COM of the biomolecule is placed as close as possible to the COM of the NP without any ABs existing inside the NP’s inner radius. In both cases, the outer bound of integration is then set such that the distance between the plane defined by (0, 0, *R*_1_^b^) and the lowermost point of the biomolecule is equal to 2 nm for consistency with previous versions of UnitedAtom and to ensure that all ABs are an adequate distance from all NPBs that they may be taken to be noninteracting.

This integration is repeated for each target orientation. By default, the angles ϕ, θ are sampled on a grid with ϕ ∈ [0°, 360°] and θ ∈ [0°, 180°]. This grid is divided into units of area 5° × 5°, with 64 points selected at random with uniform density inside each of these units. The adsorption energy is calculated for each of these 64 subsamples and averaged together to reduce artifacts and reflect uncertainty in the exact orientation of the protein. By default, this averaging employs a simple, unweighted mean, but the user can optionally enable a mode in which the local energies are averaged by their Boltzmann weights. The resulting average is then reported for the nominal lower limit of the region, that is, the output value for ϕ, θ is the average of 64 values in the region [ϕ, ϕ +5°] × [θ, θ +5°] such that the average value sampled is ϕ + 2.5°, θ + 2.5°, which should be used in postprocessing of these results. Note that these oversamples points close to either pole of the sphere with θ → 0° and θ → 180°, which must be corrected for when postprocessing results as discussed later. For postprocessing, we stress the importance of following the correct rotation procedure to avoid misinterpretation of results. Since rotation matrices in three dimensions do not commute and a rotation of 180°−θ produces a very different result to a rotation of θ, it is vital that the rotations are applied in the correct order and using the correct magnitude, *R*_*z*_(−ϕ) followed by *R*_*y*_(180°−θ).

### Configuration File

UnitedAtom is executed using the command “UnitedAtom–configuration-file = x.config”, where the configuration file instructs the program where to find all the required inputs and specifies parameters for the calculation, see Tables S1 and S2 for further information. Given the complexity of the configuration file, it is recommended that either a pre-existing template is used, with examples provided in the examples folder in the repository, or the RunUA.py script is employed to generate a suitable configuration file. This script takes as user input a folder containing target biomolecules and an NP material chosen from a predefined list, together with the radius and zeta-potential. We note that the software code interprets the supplied zeta-potential as the value of the electrostatic potential at the surface of the NP. This is not always valid and the actual surface electrostatic potential should be used if known. Further options, e.g., the temperature and ionic strength can be set as needed as described in the Supporting Information.

### Biomolecule Definition

Each biomolecule of interest is represented as a list of atomic coordinates using the PDB file format, using only CA atoms, with the three-letter residue code used to identify the specific set of interactions to employ for a bead at that location specified by the *x*/*y*/*z* coordinates (in Angstroms) fields. Further details about the specification of these files are available in the Supporting Material.

Typically, a standard protein structure file obtained from the PDB,^24^ AlphaFold,^25,26^ I-TASSER^27^ or most other sources will be directly compatible with UnitedAtom, provided it adheres to the standard PDB file format as discussed above with fixed-width columns as provided in the PDB specification. Optionally, preprocessing can be applied using the included script PreprocessProteins.py, which rotates the proteins into a standard coordinate system and replaces protonated residues as necessary, see Supporting Information for details.

**Figure 2:**
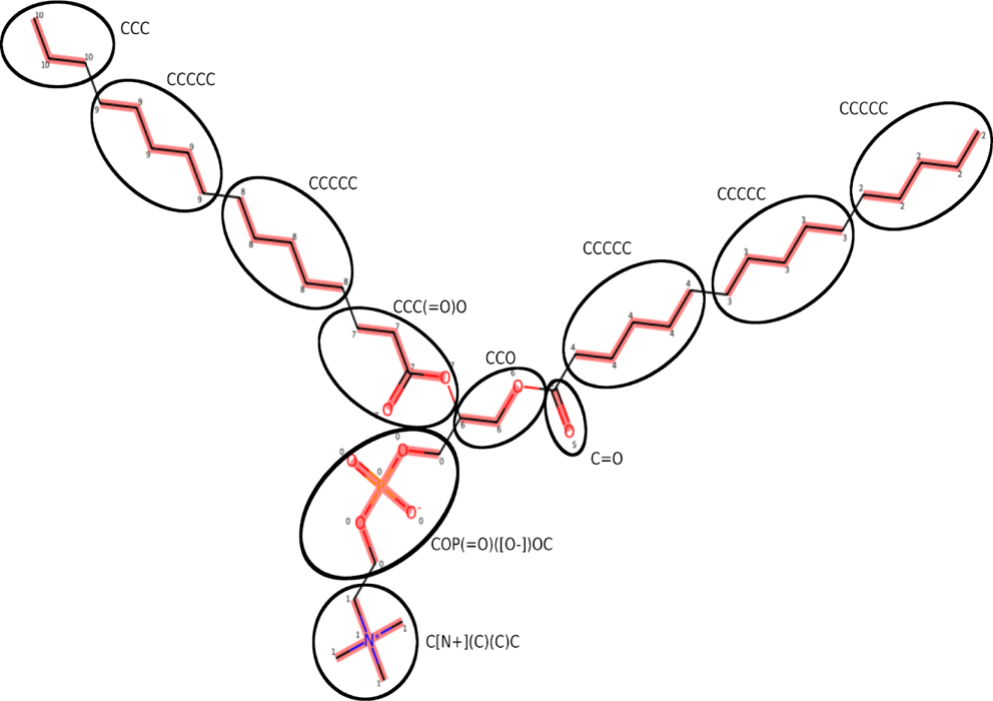
Automatically generated bead mapping for a target molecule (DPPC) using the MolToFragment.py script included in the repository, with highlighting applied to indicate the resulting fragments and manual annotation added to indicate SMILES codes for each bead. The mapping has been constrained to use only bead types for which interaction potentials are available.

For nonprotein biomolecules, a suitable CG representation is not necessarily available. Single-bead models for all the small molecules/biomolecular fragments are provided in pmfp-beads.zip, while larger molecules must be represented in terms of these available beads. For these more complex cases, we have developed a script (MolToFragment.py) to produce input compatible with UA based on matching fragments of an input molecule to predefined beads, with an example shown in Figure 2 using the “ForwardsMatching” algorithm included in this script. In brief, this script attempts to break down a target molecule into smaller fragments by matching SMILES codes of potential fragments to those corresponding to molecules which have already been parametrized. Where possible, we recommend the use of expert knowledge to produce mappings, since this may identify symmetries that the matching algorithm does not identify and this algorithm may require breaking ring structures to achieve a match. For more advanced users, additional modes are implemented for which generated splittings do not need to correspond to pre-existing fragments, e.g., the “EqualParts” method in MolToFragment.py or the BRICs method as implemented in rdkit.^28^ These will typically require the production of new interaction parameters but may produce more physically meaningful representations as these methods will not break rings. For manual generation of biomolecules, it is highly recommended to adapt existing templates to ensure that all fields are located in the correct columns; in particular, if the occupancy field is misaligned the bead will typically be assigned an occupancy of zero and thus not contribute to the binding energy.

### NP Definition

**Figure 3:**
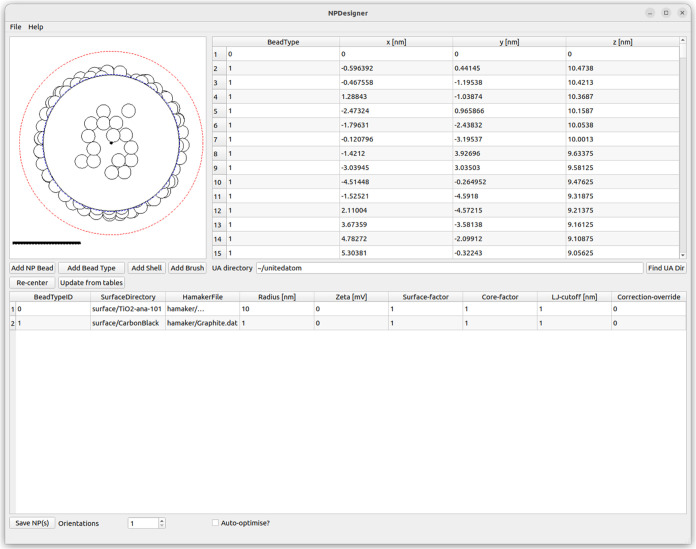
An example NP consisting of an anatase core decorated with carbon black beads produced using the NPDesigner software tool. The locations of beads are shown in the right-hand table, while the bottom table lists definitions of all bead types which have been added so far. A visualization of the NP is shown in the upper left corner, with the dashed blue line indicating the NP bounding radius at the nominal surface and the red dashed line indicating the limit at which adsorbates are assumed to be unbound.

Simple NPs consisting of a single component can be defined directly in the configuration file using the “radius” and “zeta-potential” lists together with a specified Hamaker file and surface potential directory, with UA automatically generating all combinations of these two for the material and shape in question. This further requires setting the np-shape parameter, which takes the value 1 to produce a sphere, 2 for a solid cylinder with planar-to-cylindrical potential mapping (see next section), 3 for a cube (planar mapping), 4 for tube (cylinder-to-tube mapping) and 5 for a solid cylinder (cylinder-to-cylinder mapping). Here, a tube is a hollow cylinder suitable as a model for single-wall carbon nanotubes (CNTs) while cylinders have a solid center to represent elongated NPs or multiwall CNTs. For more advanced NPs consisting of multiple components, e.g., a core with a shell or a brush, or an agglomeration of smaller NPs, the NP is defined using a specialized file format to instruct UA on the location and nature of all NP beads, with a simple example shown in Figure S7, and the np-shape option sets the global coordinate system and it is generally recommended that this be set to the spherical value. These files can be constructed manually and descriptions of the required file format are provided in the documentation in the repository, or the supplied GenerateNanoparticle.py script can be employed to generate NPs according to predefined combinations of shells and brush densities. A graphical tool NPDesigner (Figure 3) is also provided to simplify the production of common NP configurations, i.e., combinations of single beads, shells and brushes, with brushes generated one layer at a time with beads placed at locations using the algorithm presented in ref (29), with the output produced in either the .np format required for UA or in .pdb format for ease of visualization. Note that the NP is not rotated in UA itself, except for an effective rotation applied by the rotation of the biomolecule by an angle ω if this is enabled, which is equivalent to rotating the NP around the z-axis by −ω. If more complex orientations are required they must be supplied as extra .np files with the rotation applied manually, using e.g., Arvo’s algorithm to produce rotations which result in an isotropic distribution of new orientations.^30^ This algorithm is implemented in NPDesigner to allow for the production of multiple output files corresponding to the same NP in different orientations.

### Input Potentials

UA requires parameters for the interaction potentials for each biomolecule bead type with each NPB type. Three main classes of potentials are used in the UA framework, with the potential for an AB of type *i* with an NPB of type *n* given by,
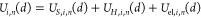
5where *d* is the distance of closest approach between the beads, *U*_S_ is a tabulated short-range (surface) potential corresponding to the interaction between the AB and the surface of the NP, *U*_H_ is a Hamaker-like (integrated vdW) potential and *U*_el_ is an electrostatic potential. The tabulated short-range potential *U*_S_ must be provided for each NP material and AB by specifying a folder for that material containing a set of files XXX.dat, where XXX is the three-letter code associated with that AB and must be consistent with the definition used in the configuration file and biomolecule structure file to ensure that UA assigns the correct potential to each bead. Each surface potential file should contain a comma-separated table of values for the potential (units kJ·mol^–1^) as a function of the distance of the center of the bead to the surface of the NP (units nm) as shown in Figure S8. We note that some older files use a fixed-width file format, which remains functional within UA for backward compatibility but should be considered deprecated in favor of comma-separated files.

The surface potential file is most frequently a PMF obtained via metadynamics, umbrella sampling or ML methods, but can in principle correspond to any distance-dependent potential to apply representing the interaction between a given AB and a particular nanomaterial surface. All tabulated potentials supplied in the repository correspond to PMFs obtained either via atomistic molecular dynamics simulations with enhanced sampling (metadynamics or umbrella sampling) or the output from a machine-learning model trained on atomistic PMFs. We note, however, that in principle these can be computed through other sources and UA is largely agnostic to the means of computation. Due to the high computational cost of generating these potentials, they are typically are computed for each AB of interest (e.g., the set of AA-SCA beads) to a particular NP topography (e.g., a planar surface or a cylinder of predetermined radius) of a specific material. To allow these to be used for NPs of the same chemical composition but different geometries or radii, a correction function is applied to map these to the expected potential generated by the actual NP of interest, e.g., mapping from a planar configuration to a spherical NP of the given radius. This correction function is generated under the approximation that the main contribution to the surface potential arises from the 1/*r*^6^ vdW term, such that the ratio of the tabulated potential generated by a volume *v*_1_ to that generated by the volume *v*_2_ is approximately equal to the ratio of the *r*^–6^ potential integrated over these two volumes,
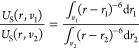
6where the integration runs over all points *r*_*i*_ in the NP volume region *v*_*i*_ included in the calculation of the surface potential, generally taken to be the volume of the NP within the typical LJ cutoff for that force field.^9,31,32^ This correction is implemented for the plane-to-sphere geometry as originally discussed in^9^ and has been extended to plane-to-cylinder, cylinder-to-tube, and cylinder-to-cylinder geometries. Although this is an approximation, it has been shown that e.g., the cylinder to plane transformation does not significantly alter the form of a PMF,^15^ which we attribute to the relatively short-range nature of the interactions involved. Alternatively, no correction can be applied, which is required for PMFs generated for small polymer beads. For PMFs generated for other geometries, it is recommended to manually map these to a planar configuration such that UA can automatically remap them to the target configuration as required. At present, the PMF is a function of only the distance of the bead to the surface and does not account for the orientation of the molecule or internal degrees of freedom. We therefore recommend that PMFs represent small, reasonably rigid sections of biomolecules to minimize the errors introduced by this.

The tabulated short-range potential is assumed to correspond to only a fraction of the total volume of the NP close to the ABs. To account for the rest of the NP, UA generates a long-range Hamaker-like potential *U*_H_ corresponding to the integration of the vdW potential over the volume of the NP and AB.^31,32^ Unlike the traditional Hamaker approach, the integration is performed only over elements of each bead separated by a distance greater than a cutoff distance *r*_c_ and is not limited to sphere–sphere interactions only, with the generic integral given by,
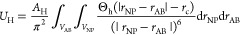
7where *A*_H_ is the Hamaker constant for that particular AB–NPB pair interacting through water,^31^ Θ_h_(*x*) is the Heaviside theta function, used to set the integral to zero within the exclusion region, *r*_c_ is the cutoff distance at which the interaction is covered in the PMF (assumed to be equal to the LJ cutoff in the metadynamics simulation) *r*_NP_ is a point in the NP, *r*_AB_ a point in the AB, and the integration runs over all pairs of points. This produces a function that smoothly switches between different regimes as necessary without a discontinuity at distances of *r*_c_ which would be introduced if the interaction is simply switched on once the bead center is sufficiently far from the NP. For a spherical NP at long-range eq 7 reduces to the standard Hamaker expression, whereas different results are obtained for cylindrical geometries or for configurations at close range to avoid double-counting elements of the adsorbate or NP beads. For cylindrical geometries, this expression is evaluated partially numerically due to the lack of a closed-form analytical result. The required Hamaker constants are supplied in an input Hamaker file for each NP material, consisting of one line per AB type, with the required input format specified in src/HamakerFile.h as depicted in Figure S9. These can be computed through Lifshitz theory^32^ or through summation of force field parameters as implemented in either the Enalos Hamaker tool^33,34^ or the scripts supplied in ref (19), with the latter used to produce Hamaker constants matching the materials with ML surface potentials included in the repository. This also requires the radius for the bead as set in the configuration file, where the radius is typically calculated from force field parameters or experimental data as discussed later.

The third contribution is an electrostatic potential, for which we employ the Debye–Hückel approximation to the Poisson–Boltzmann equation to represent the effects of electrostatic screening while allowing for simple analytical expressions for all geometries to be determined. The resulting potentials are defined by the Debye length κ^–1^ as specified in the configuration file, the charge of the AB *q*_*i*_, the surface potential ψ_0_, and the shape of each NPB. For a spherical NPB, the resulting potential is given by,
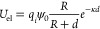
8cylindrical by,
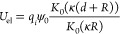
9and planar by,
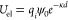
10noting that for all three we model the AB as a point particle such that *d* is the distance from the surface of the NPB to the center of the AB. An expression for finite cubes based on an expansion in terms of spherical harmonics is implemented in the code but is employed only when the size of the NP is on the same order of magnitude as the Debye length, which is generally less than 1 nm and so the planar potential is typically acceptable. For historical reasons, the value of the surface electrostatic potential is referred to as the zeta-potential and a Bjerrum-length parameter is also read in from the configuration file. The Bjerrum length is unused except in certain special cases discussed further in the UA documentation; in typical operation, this parameter can simply be left at its default value as it does not enter into the electrostatic calculations. We also note that many PMFs for charged surfaces already include the effects of the charge–charge interaction and, since the Debye length in UA is typically on the order of 1 nm, the majority of the surface charge is already accounted for by the PMF. Thus, in some cases, it may be more accurate to set the electrostatic surface potential to 0 mV to avoid double-counting the charge–charge interactions, unless it is known that the PMFs did not include a charge, e.g., the set of zerovalent metal PMFs or if the electrostatic potential is used to offset the charge interaction already factored into the PMF to produce a different overall surface charge.

### Output

**Figure 4:**
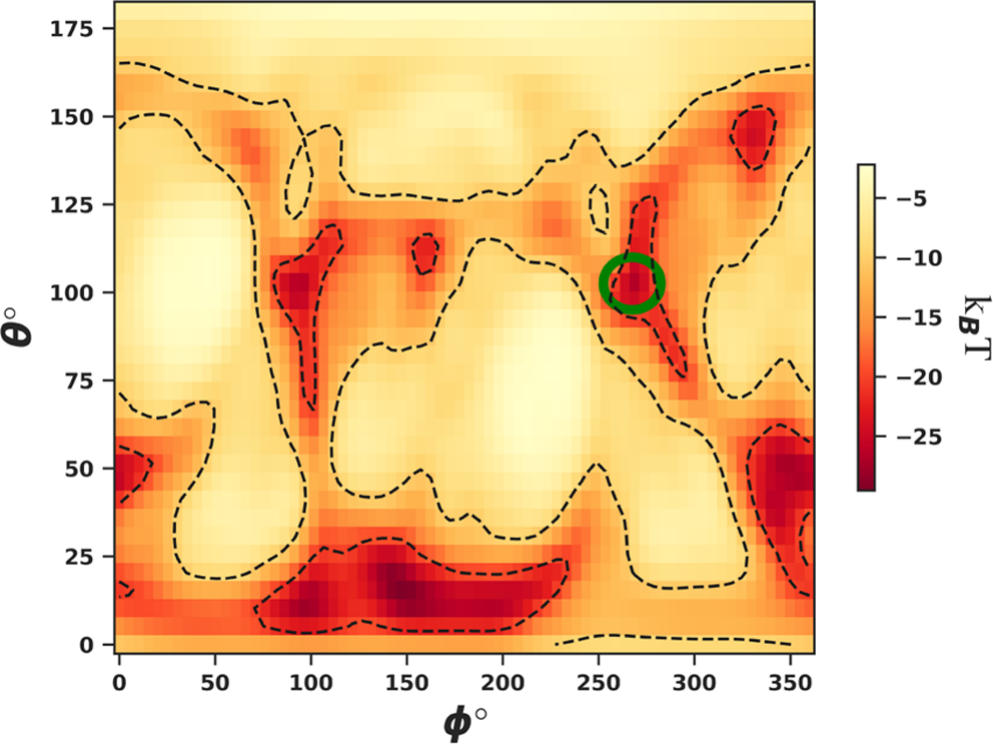
An example heatmap plot of binding energies produced for bovine serum albumin (PDB code 3V03) to a silver NP (Ag (100), *R* = 27 nm, surface potential −31 mV). The location of the most favorable protein orientation is marked with a green ring at ϕ = 267.5°, θ = 102.5°.

**Figure 5:**
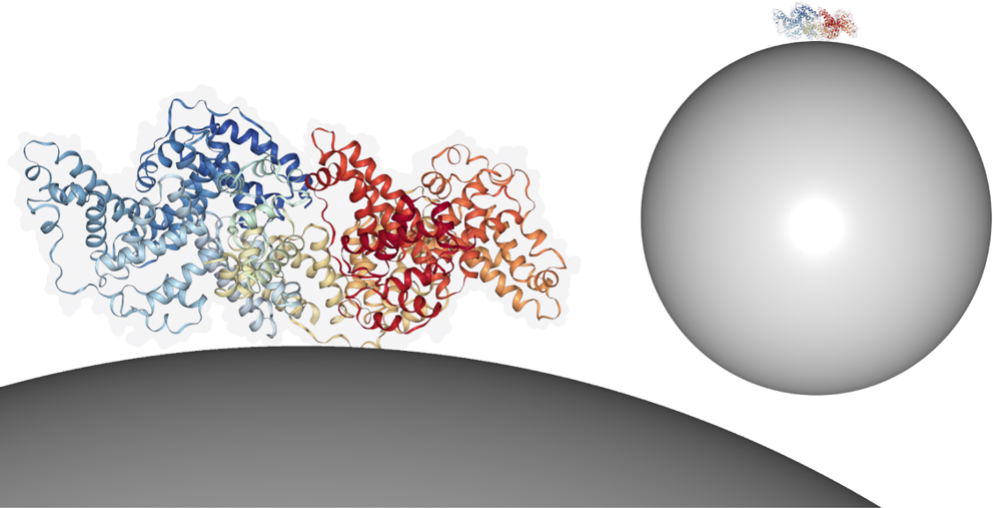
An example of the protein–NP complex produced by postprocessing the results of a UA calculation for bovine serum albumin (PDB: 3V03) to a silver NP (Ag (100), *R* = 27 nm, surface potential −31 mV) using the VisualizeUAResults.ipynb script. The conformation shown is the energetically most favorable orientation of the protein. The inset shows the entire complex while the main figure provides a cropped region to show finer details of the protein.

The main output from a UA run is a datafile with an automatically generated filename of the form “biomolecule_radius_zeta.uam” for each NP–biomolecule pair, stored in the designated output folder defined in the configuration file. This datafile contains a table of values mapping each orientational sampling range (given as left-hand edges for ϕ, θ and the fixed value for ω) to a local average of the adsorption energy (provided in units *k*_B_*T* and kJ·mol^–1^), the standard deviation of adsorption energies in this interval, mean-first-passage-times (if enabled, else this field contains the value −1), the distance between the nominal surface of the NP and the center of the closest AB, and the average number of residues in close-contact (at a surface to AB center distance of under 0.5 nm) in that range of orientations. Typically, this datafile is postprocessed to provide further results. A very common application is the generation of a heatmap plot as in Figure 4 to highlight the general adsorption affinity of the biomolecule to the NP and identify strongly adsorbing orientations and plotting the most favorable conformation as in Figure 5 (both generated using the script provided in tools/VisualizeUAResults.ipynb, see Supporting Information).

To provide an immediate assessment of the affinity of a given biomolecule to an NP, the binding energy is averaged over all orientations according to a given weighting scheme, e.g., the simple average:
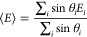
11or Boltzmann-weighted average,
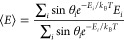
12In both of the above, the index *i* refers to a specific orientation with associated angles θ_*i*_, ϕ_*i*_ and adsorption energy *E*_*i*_ = *E*(θ_*i*_, ϕ_*i*_). Note that UA output files contain the left-hand edge for θ and thus these must be offset by 2.5° to obtain the central bin, then converted to radians before calculating sin θ. Of these two averages, the simple average can be thought of as the affinity of an adsorbate which is at a random orientation with respect to the surface of the NP, i.e., during the initial stage of the corona formation. The Boltzmann average, meanwhile, is more strongly weighted toward orientations with high binding affinity, and so reflects the thermal equilibrium achieved in the later stages of corona formation. A script ExtractBindingEnergies.py is provided to calculate these averages for convenience and produces a table of energies for a given set of input folders for comparison across biomolecules and further use. In certain cases, these averages must be computed taking into account the fact the protein can bind to multiple different surface types, e.g., different crystal facets, Janus particles, or if multiple values of ω have been sampled for, e.g., CNTs. This is achieved by generalizing the above expressions to include an additional weighting term in the numerator and denominator, *w*_*j*_, to reflect the abundance of that particular surface,
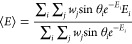
13as demonstrated in the MultiSurfaceAverage.py script included in the repository. Further pre- and postprocessing scripts are documented in the Supporting Information including advanced visualization tools.

## CoronaKMC: Corona Prediction via Kinetic Monte Carlo

The adsorption affinity of a biomolecule to an NP is not necessarily predictive of its abundance in the corona, especially when there is competition between multiple adsorbing species or orientations of the same species. A large protein may adsorb very strongly but exist in such vanishingly low concentrations compared to other potential adsorbates that its overall abundance remains low, or it may be out-competed by biomolecules that individually adsorb less strongly but occupy a smaller area such that the total energy is more favorable by adsorbing a large number of these, or even be out-competed by another absorbate which binds even more strongly. If, however, no other adsorbates are present then this large protein will then be a major component of the corona. Consequently, a prediction of the corona content must take into account this competition between all adsorbates present.

A very simple first-order prediction of the corona content may be obtained using the mean-field approximation.^35,36^ Given a set of adsorbates *i* with adsorption free energies *E*_*i*_, concentrations *c*_*i*_, and *n*_*i*_ available binding sites on the surface of the NP, where *n*_*i*_ is inversely proportionate to the cross-sectional area of the adsorbate, the number abundances are approximated by,
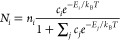
14This simple expression neglects a number of factors, chiefly, it allows for completely efficient packing of adsorbates onto the surface of the NP and assumes adsorbates can deform to an arbitrary degree. We have previously demonstrated a hard-sphere model of corona formation which overcomes these limitations.^12,14^ In this section, we discuss the implementation of the KMC method for evaluating the corona formation as integrated into this package. In brief, this script simulates the sequential adsorption and desorption of adsorbates to the surface of an NP, taking into account factors such as the bulk concentration and availability of free surface area on the NP for binding to take place. The NP is assumed to be a single bead of either spherical, cylindrical or planar geometry with adsorption occurring isotropically across its surface. Thus, if an NP consists of multiple surfaces such as a Wulff structure or a Janus particle, we recommend that a separate simulation is run for each surface type of interest and the total numbers of adsorbed proteins calculated as a weighted sum over all surface types.

### Input

The most important input to a CoronaKMC run is a list of all potential adsorbates, defining their effective size, concentration in the bulk, and rate constants for adsorption and desorption. In simple cases, this file can be manually constructed. In general, however, the BuildCoronaParams script should be employed to automate the conversion of .uam output and .pdb structures to the required input format. This script takes as input a list of biomolecules and their number concentrations in units mol/L, finds matching structures and .uam binding energy tables and computes rate constants and adsorption areas for each orientation of the biomolecule.^13^ The output is saved in the structure shown in Figure S10, in which each orientation of a given biomolecule is assigned an individual identification and set of rate constants.

Once an input set has been generated, the script is run using Python 3. In addition to the list of adsorbates, further options can be specified as command-line arguments when running CoronaKMC to further control the simulation parameters. These options are described in more detail in the documentation and typically enable features such as manual control over the boundary conditions and coordinate system, whether the simulation should run in time-resolved (kinetic Monte Carlo) or steady-state conditions (classic Monte Carlo), specification of the amount of simulated time for which the program should run, the use of an algorithm to accelerate the simulation by identification of quasi-equilibrated processes,^37^ the activation of the optional “displacement mode” instead of “standard mode” (discussed later), and other parameters. During the simulation, events are generated corresponding to the adsorption or desorption of adsorbates. Adsorption events correspond to the selection of a potential adsorbate with a probability proportional to the rate at which it collides with the NP and the generation of a random position on the surface of the NP. By default, it is assumed that these rates are determined using the provided BuildCoronaParams.py and correspond to physically realistic values, such that the program simulates the full time dynamics. If the optional steady-state mode is enabled, both *k*_a_ and *k*_d_ for each adsorbate are rescaled while keeping their ratio fixed. This rescaling effectively normalizes all proteins to collide with the NP at approximately the same rate while adjusting their desorption rate equivalently to produce the same equilibrium state^12^ without the requirement to simulate the full evolution of the corona and thus significantly reduce the computational time required to compare to experimental results. This is valid only in standard mode, but if this option is set then the simulation temporarily employs displacement mode to further accelerate convergence, see Supporting Information for more details.

The acceptance of an adsorbate depends on the selected mode. In standard mode, the adsorbate is automatically accepted if there is sufficient room for it to contact the NP without the projection of this adsorbate onto the surface overlapping with the projection of a pre-existing adsorbate, else it is rejected. In this mode, a small, weakly binding adsorbate may block the adsorption of a large, strongly binding adsorbate, which may not be physically realistic for a given system. Thus, if the optional “displacement mode” is activated, then the adsorbate is accepted with a probability of *e*^–Δ*E*/*k*_B_*T*^/(1 + *e*^–Δ*E*/*k*_B_*T*^), where Δ*E* is the difference between the binding energy of the incoming adsorbate and the sum of the binding energies for all currently adsorbed particles which would overlap with the new adsorbate. If the adsorbate is accepted, all overlapping preadsorbed particles are removed to make room for the new one. Note that water is not explicitly included in these simulations unless it is manually added as an adsorbate. In standard mode, water is implicitly assumed to be accounted for in the provided rate constants. This is indeed the case for adsorption energies computed using UnitedAtom using the default procedure, since the input potentials (particularly PMFs) are computed in the presence of water and typically feature repulsive barriers corresponding to the presence of water. The acceptance probability in displacement mode given above is consistent with a model of implicit water in which water adsorbs with a reference energy of 0 kJ·mol^–1^ and it is assumed that regions of the NP without explicit adsorbates are covered in water. Thus, the adsorbate binding energies should be calculated with respect to this reference value, i.e., to include the requirement to displace water for adsorption. As mentioned above, this is already accounted for in UnitedAtom. Note that the acceptance probability in displacement mode is defined such that adsorption which does not change the overall energy is allowed 50% of the time such that both outcomes occur with equal probability. Desorption occurs with a probability dependent on the desorption rate constant; this value is scaled slightly in displacement mode to ensure that the ratio *k*_a_/*k*_d_ remains fixed due to the decrease in adsorption for weakly adsorbing biomolecules.

### Output

**Figure 6:**
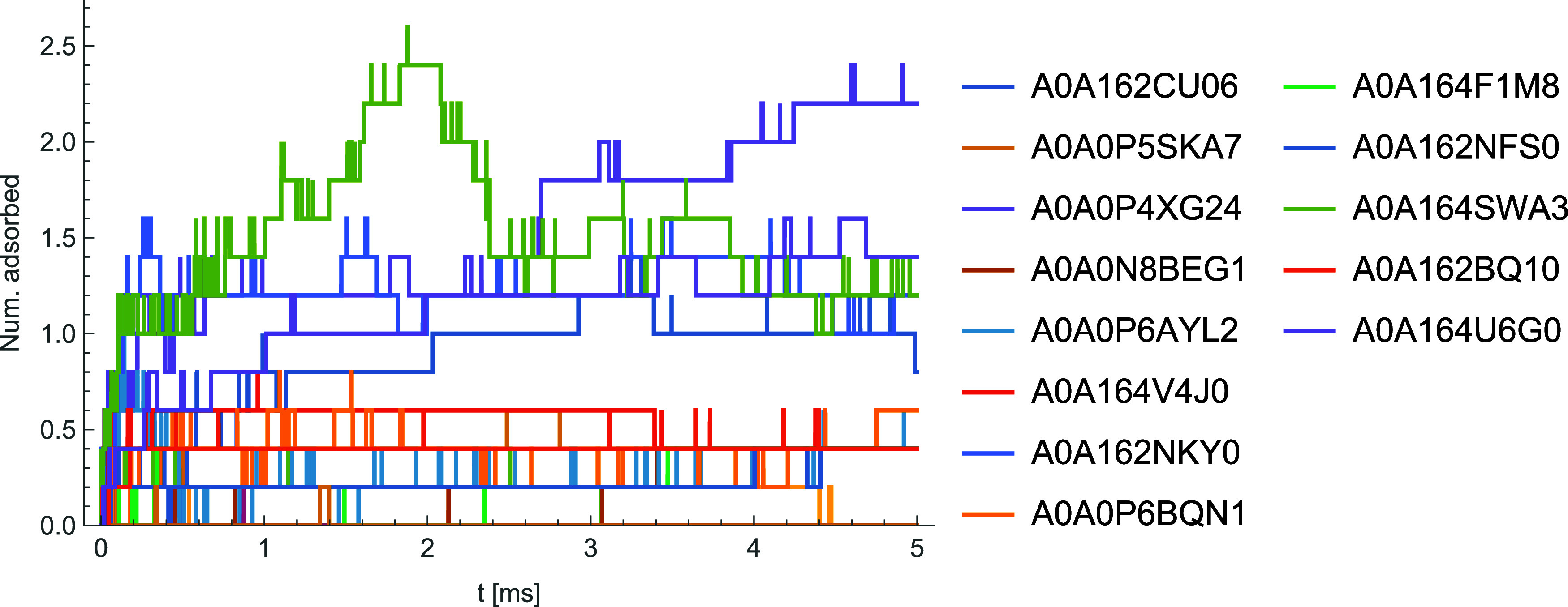
Time-evolution of the predicted corona for a set of 20 proteins (Table 1) on a 5 nm gold (100) surface, averaging over five simulations. For clarity, only proteins still in the corona at *t* = 5 ms have an entry shown in the legend.

During the runtime of a simulation, the number of each class of adsorbate (summed over different orientations of the same species) is displayed on-screen at predefined intervals, with the same data saved to text files for further use such as plotting the evolution of the corona as shown in Figure 6. At the end of a simulation, coordinates for the final corona composition are saved out including the exact identity of each adsorbate to allow for identification of orientations which are present in the corona and for visualization purposes if necessary. Two such files are generated: one with a .kmc extension, which contains the data in the internal coordinate system and the adsorbate rate constants, and a plain text .txt file with “finalcoords” in the filename, which contains the adsorbate name and Cartesian coordinates. An example script tools/CoronaKMCtoVMD.py is provided to convert an output .kmc file for a spherical NP to a .tcl script which can be run in VMD to produce a simple visualization of the corona. More advanced visualization can be achieved using the BuildCoronaCoords.py script, which produces an output .pdb file containing coordinates based on the atomistic coordinates for all molecules found in the corona in their correct orientations and locations.

## NPCoronaPredict: End-to-End Prediction

In many cases, the same set of biomolecules must be tested against a variety of NPs under essentially identical conditions. To facilitate this, we have developed a wrapper script NPCoronaPredict.py (formerly PrepareKMCInput.py) to automate running the UA to BuildCoronaParams to CoronaKMC pipeline for simple NPs. This script takes as input the list of biomolecules of interest together with their concentrations and automates setting up and performing each step of the calculations, including fetching protein structures from the AlphaFold repository if possible for adsorbate names which correspond to a valid UniProt ID. As additional input, it takes the NP size, shape, and material, along with any other parameters to pass to UA or CoronaKMC as necessary. The NPCoronaPredict-GUI tool provides a simplified wrapper to this script to enable corona prediction purely via the graphical interface.

**Figure 7:**
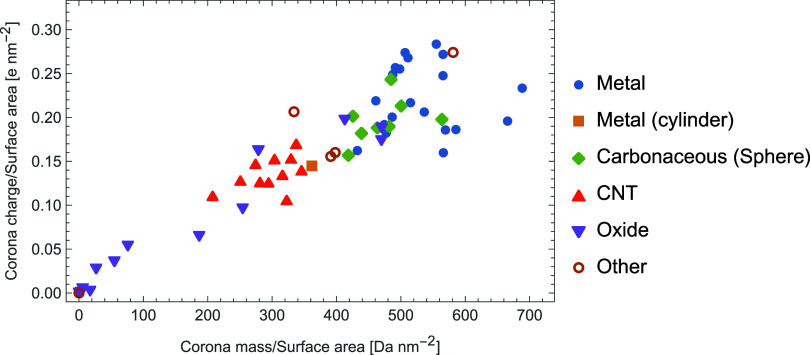
A plot of predicted surface-area normalized corona mass and charge for a variety of nanomaterials immersed in a medium of 20 proteins. The proteins are selected from the *Daphnia magna* proteome based on a cluster analysis to demonstrate the use of the pipeline to assign a simple low-dimensional representation to arbitrary nanomaterials broadly matching their chemical classes (metallic, carbonaceous, metal/semimetal oxide), with the other category including semiconductors and metals with organic ligands attached. An exponential smoothing with a time constant of 0.1 ms has been applied to reduce noise.

As a demonstration of the use of this automated scanning, we have performed corona simulations for a trial solution of 20 proteins selected from the proteome for *Daphnia magna*, using the AlphaFold structures for these and selecting proteins based on clustering of their properties. These input descriptors are selected from a large set of descriptors calculated via PEPSTATS, a modification of PEPSTATS to produce properties only for surface AAs, and additional descriptors related to the structure of the protein, with the k-means algorithm used to select 20 proteins. The resulting proteins and the concentrations assigned are given in Table 1, where concentrations are chosen such that the mass concentration of each protein is equal. We stress that this does not correspond to a real experiment, but is done primarily as a demonstration of the flexibility of the pipeline to handle complex mixtures of potential adsorbates and of postprocessing techniques which can be used on the resulting data. Corona predictions have been performed for a set of 58 materials broadly separable into three main groups: metallic, carbonaceous and metal/semimetal oxides, with the remaining materials (CdSe, gold with organic ligands, MoS_2_) classified as “other”. For consistency, we employ the ML PMFs, use a radius of 5 nm for all NPs, taking spherical NPs except for CNTs and a cylindrical platinum (001) NP included to enable a comparison to the spherical form. Simulations are performed with an internal averaging over five instances of each NP to reduce statistical artifacts due to the low levels of adsorbed proteins at this size. The GetCoronaStats.py script included in the repository is employed to postprocess these by calculating corona-totaled values of each protein descriptor *X* by calculating ⟨*X*⟩ = ∑_*i*_*x*_*i*_*N*_*i*_/∑_*i*_*N*_*i*_, where *x*_*i*_ is the value of *X* for adsorbate *i* and *N*_*i*_ is the number of instances of that adsorbate in the corona. We use these to describe each material in terms of a pair of simple descriptors: the total mass and charge of adsorbates, normalized to the surface area of the NP to ensure a fair comparison between spheres and cylinders. The resulting values after 5 ms of simulation time are plotted in Figure 7 to demonstrate the use of this pipeline in performing a rapid categorization of nanomaterials in a given medium. It can clearly be seen that the three main groups specified above form clusters in different regions of the chart, although with some overlap between these, and that cylindrical NPs typically exhibit less adsorbed mass per unit surface area than spherical forms. We attribute this latter effect to the difference in packing efficiency around spheres compared to cylinders.^12^

**Table 1:** Proteins Selected from the *Daphnia magna* Proteome Based on k-Means Clustering of PEPSTAT and Other Descriptors

ID	concentration [μM]	mass [kDa]	charge [e]
A0A162CU06	3.76	13.31	4.5
A0A0P5SKA7	1.20	41.56	12.5
A0A0P5LW78	2.29	21.83	–1.5
A0A164 KXJ8	0.48	103.34	9.5
A0A0P4XG24	5.23	9.57	10.5
A0A0N8BEG1	1.49	33.64	–4.5
A0A0P6AYL2	7.20	6.95	3.5
A0A0P5SS69	2.02	24.80	3.5
A0A0P5WS26	1.72	29.01	6.5
A0A164Z8Z4	0.51	98.27	0.0
A0A164 V4J0	3.76	13.31	9.5
A0A162NKY0	4.82	10.38	2.0
A0A0P6BQN1	5.33	9.37	11.0
A0A164Z4N7	0.79	63.42	0.0
A0A164F1M8	1.21	41.37	8.0
A0A162NFS0	0.87	57.50	0.0
A0A164 × 841	1.37	36.45	8.0
A0A164SWA3	8.79	5.69	4.0
A0A162BQ10	3.55	14.08	–6.0
A0A164U6G0	1.93	25.90	5.5

### The NPCoronaPredict–GUI Graphical Interface

**Figure 8:**
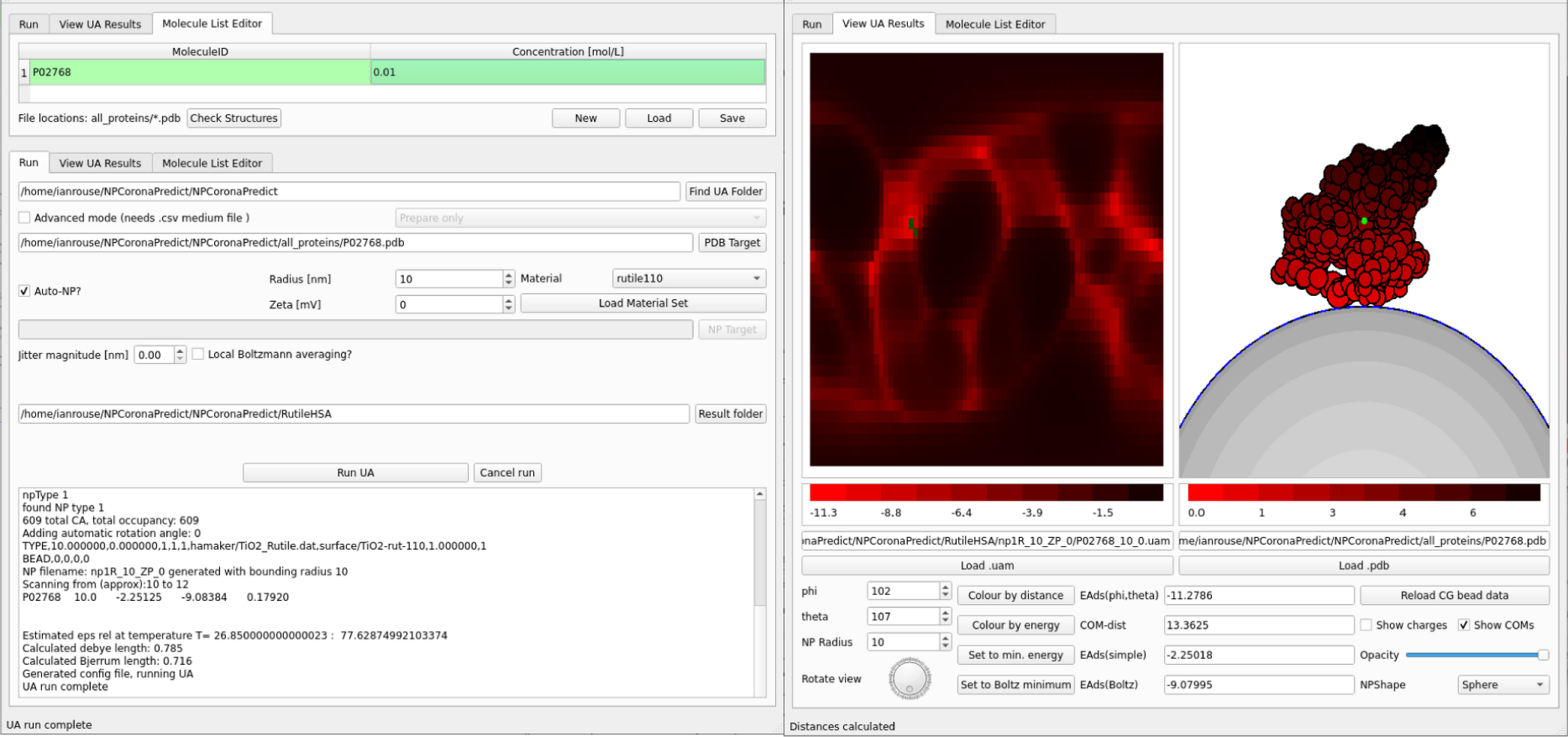
A demonstration of the NPCoronaPredict-GUI interface for performing NP–protein binding energy calculations using a simplified set of options. The first panel (top left) shows the interface for automatically downloading protein structures based on their ID. The second (bottom left) shows the setup and output of computation for a single protein–NP pair (here human serum albumin to a rutile NP). The third (right) shows the results visualized as a heatmap and schematic view of the favored orientation.

To assist new users and to allow for rapid interpretation of results, we have designed a simplified GUI named NPCoronaPredict-GUI to streamline some of the more common uses for UA as shown in Figure 8. This GUI combines three main tools to simplify the potentially complex procedure. First, a list of biomolecules of interest can be edited on the “Molecule List Editor” tab and structures for these found if needed. Structures defined here are automatically retrieved if needed either from the RCSB PDB^24^ if they are given a label in the form “PDB-X”, where X is the PDB ID for that protein, or from the AlphaFold database^25,26^ for identifiers of the form “AFDB-X”, where X is a UniProt ID. Note that structures can be manually provided if needed and will only be fetched remotely if none is located and if the user requests this. The main functionality is located on the “Run” tab, which allows the user to generate basic NPs or select an output from NPDesigner, and select a protein (or list of biomolecules) of interest. The GUI can then be used to call the UnitedAtom executable via the main NPCoronaPredict script and show the results as these are computed. For simplicity, this uses the steady-state options for CoronaKMC to attempt to provide the final corona as quickly as possible without the user needing to run the application for an extended period of time. Once a run is complete, the generated data can be visualized as a heatmap and the location of the protein relative to the NP is visualized. The user may furthermore perform a corona prediction for a given list of proteins on a target NP by activating the relevant settings. For reasons of computational efficiency this is set to produce the steady-state rather than a full time-resolved corona prediction under the assumption this is more experimentally relevant. We stress that by design, this interface does not incorporate the full functionality of the NPCoronaPredict package, but provides a simplified experience for new users or those less familiar with command-line operations.

## Material Library

To enable a wide application of the software discussed here, PMFs and Hamaker constants have been computed and included in the repository for a wide range of materials, with a particular focus on the adsorption of AA/SCAs to these surfaces. In this section, we present an overview of the calculations used to parametrize these interactions and descriptions of the available surfaces. Calculations performed using these potentials should cite the original works.

### PMFs

Tabulated PMFs for sets of biomolecular fragments have been computed using atomistic metadynamics or umbrella sampling simulations for a range of materials: gold (100, 110, 111), silver (100, 110, 111), aluminium, iron, carbon nanotubes (pristine and modified with a range of functionalizations), graphene (1, 2, 3 layers), graphene oxide, reduced graphene oxide, amorphous carbon (three morphologies), titanium dioxide (two rutile, two anatase surfaces), silica (amorphous and quartz), iron oxide, cadmium selenide. We also supply PMFs for a PEG trimer to allow the construction of brushes but note this requires additional configuration or the use of the PEG-Slab potentials derived from this, see Supporting Information.^11^ Due to the differing availability of force fields and computational methods (especially metadynamics settings, ionic strength and species, and choice of fragments) used, these PMFs differ slightly in terms of coverage of small molecules and details such as the appropriate LJ cutoff to employ. A summary of the PMFs provided in the library is presented in Table S3. For the majority of these materials and including a range of further materials of interest, a previously developed ML approach has been employed to produce PMFs for an extended bead set and is trained on the PMFs obtained via metadynamics as described above.^18^ For full details we refer interested readers to this prior work, which provides information on the methodology, training set, and validation method. We have made some minor updates to the methodology employed, primarily a few adjustments to the network architecture, the inclusion of additional materials in the training set (zinc sulfide,^38^ zinc oxide,^39^ FCC copper, aluminum and iron^14,40,41^), and PMFs produced for both SCAs and full AAs for rutile using an alternative force field.^42,43^ We have also made some changes in the input potentials used, with these changes made available in the model repository.^19^ The most significant correction was the reidentification of a set of PMFs for the Au (100) surface as having been generated for full AAs rather than SCAs, which previously led to a decrease in the model accuracy. The final PMFs are produced “in the style of” the methodology used for the TiO_2_ and carbonaceous materials for all materials to produce a uniform standard to facilitate comparisons across materials. Likewise, we employ the same convention for the small molecule bead types to allow for comparison between nanomaterials, that is, we generate PMFs for the SCAs except for full-molecule models for proline and glycine. A naming convention for the AA beads is employed to match the standard expected by UA such that the three-letter AA codes correspond to the PMF of the SCA while the Hamaker constant is calculated for the full AA; all other beads use the same structure for both PMF and Hamaker constant. A preaveraged HIS bead, consisting of weighted averages of the PMFs and Hamaker constants for HIE, HID, and HIP, is provided for pH 7 with a range of individual charge variants for other AAs also produced. The additional materials include Ag (332) and (322), Pt (001), Ce (001), a set of weathered Au surfaces with varying percentages of surface atoms removed, Au decorated with PE and PEG brushes, hydroxyapatite (at a range of pH values and surface indices), stainless steel, tricalcium silicate, CaO, MoS, Al_2_O_3_, and Cr_2_O_3_, and a range of clay materials. For all of these additional surfaces, force fields and structures were obtained using the Charmm-GUI nanomaterial modeler^44^ and the InterfaceFF force field,^45^ with the exception for stainless steel for which the structure was obtained from ref (46).

### Long-Range Interaction Parameters

Special attention must be paid to the computation of the parameters required to evaluate the Hamaker-like long-range potentials, these being the bead radius and Hamaker constant for each surface-bead pair. In the following sections, we discuss in more detail how these parameters have been calculated for the materials and chemicals provided in the repository. We note, however, that as with the PMFs, the user is free to supply their own calculated values.

#### Bead Radii

When the standard bead set is used, the radius is calculated according to the methodology in ref (47), which is calibrated to reproduce experimental protein densities from the vdW radius and coordinates of each AA. For the extended bead set which must account for arbitrary molecules, no equivalent procedure is available and we instead estimate the volume occupied by the molecule based on the LJ parameters of the atomistic representation of this molecule. We have developed two methodologies to do so. The first models each constituent atom as a sphere of radius σ_*i*_/2 at the location given by the coordinates used in the input structure for that molecule, and generates points on the surface of each atom, with the convex-hull method used to select the points generating the outer surface of the molecule. The total volume occupied by this surface is then computed and the radii of the sphere with an equivalent volume are recorded. In the second approach, the orientation-averaged self-interaction via the LJ potential between the molecule with a copy of itself is recorded as a function of the distance between the centers of mass of the pair. We approximate that the effective radius of the bead is then one-half of the distance of the first zero-crossing at which the potential switches from repulsive to attractive, by analogy to the standard LJ potential. This latter methodology provides results which are generally consistent with the values from ref,^47^ with a linear least-squares fit providing *R*^2^ = 0.82 when the outlier histidine is excluded for consistency with that work. In Supplementary Table S4 we present the results for both of these methodologies compared to the values in ref (47) and those used in ref (48) and extracted from solution-phase values from ref (49). The convex-hull radii can be seen to be an extremely good reproduction of the values found for solution-phase AAs, which are likewise quite close to those found for AAs in proteins.^49^ Thus, we recommend the use of the convex-hull radii and have supplied these in the repository.

#### Hamaker Constants

The Hamaker constants used as the overall energy scale for a given potential can be rigorously computed using Lifshitz theory based on optical constants for the NP and molecule.^32^ This constant is denoted *A*_132_, where 1 is taken to be the AA, 2 the NP and 3 the medium, typically water, and is given by the sum of a zero-frequency term *A*_132_(0) defined by,
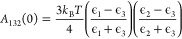
15and a high-frequency term,
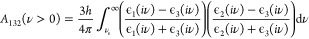
16In the above, ϵ_*i*_ = ϵ_*i*_(0) is the dielectric permittivity at low frequency, while ϵ(*iν*) is the permittivity at imaginary frequencies and ν_s_ = 2*πk*_B_*T*/*h*. For dielectric components (solvent, nonmetallic NPs, ABs) we approximate,
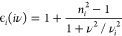
17where ν_*i*_ is the electronic absorption frequency in the UV and *n*_*i*_ is the refractive index at visible wavelengths. For metal components, we instead employ,
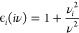
18where ν_*i*_ is the free electron gas (plasma) frequency for that metal with ϵ_*i*_(0) → ∞ and the refractive index not defined. We assume all ABs are dielectric and that the solvent is water, such that we only need expressions for dielectric and metallic NPs. For a dielectric NP and approximating that all three absorption frequencies are all equal to the same value denoted ν_e_, *A*_132_(ν > 0) is approximately given by,
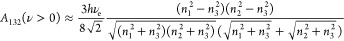
19

For a metallic NP, we numerically integrate eq 16 using the appropriate expressions for each dielectric permittivity, allowing the absorption or plasma frequency to differ for each component. These expressions are implemented in the preprocessing script CalcLifschitzHamaker.ipynb as described in the Supporting Information. Typically, the required optical constants must be found in databases of experimental results or computed from first principles, which may not be possible or be extremely time-consuming. The estimation of Hamaker constants from first principles can be also achieved through the calculation of the LJ constant *C*_6_.^50,51^ However, this method is also time-consuming and cannot be straightforwardly automated as is required here. In the case where optical data is not available, we instead approximately extract the Hamaker constant from force field parameters for the species in question. This methodology is implemented in the Enalos Hamaker Constant Tool (EHCT),^33,34^ which requires only empirical formulas and densities as input. An automated routine to perform the calculation is also implemented in the PMFPredictor Toolkit.^19^ In this method, we compute the vacuum self-interaction Hamaker constant for a given AB by summation over force field parameters similar to the method in the EHCT,
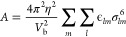
20with ϵ*_lm_*, σ*_lm_* computed using combination rules, η = 0.64 is the packing density for random close-packed spheres, and *V*_b_ is the approximate volume per molecule, with this volume set equal to the convex-hull volume as discussed in the previous section, such that *V*_b_/η represents the volume occupied by the molecule in the condensed phase. This procedure typically produces values in the range 0.1–1.0 × 10^–20^ J, in agreement with the range expected for Hamaker constants for organic molecules. We note that the computed value for water of 6.8 × 10^–20^ J is slightly larger than the values presented in ref^32^ of (3.7–5.5) × 10^–20^ J, but within an acceptable error given the generally small contribution from the Hamaker potential for beads of this size. We employ the same approach to generate Hamaker constants for each surface structure, with the exception that since the coordinates for these are assumed to already be in the solid phase we set η = 1. In principle, combining relations can then be used to produce the Hamaker constant describing the interaction between each bead and the surface in a medium *w*,
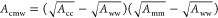
21These relations, however, are known to be inaccurate when the medium is water, as is the assumed case here.^32^ We find in particular that for metallic NPs, for which *A*_mm_ is large, the results are highly dependent on the relative values of *A*_cc_, *A*_ww_ since this may easily produce strongly positive or negative values with slight variations in *A*_cc_. Thus, we instead use the Lifshitz model as described above by finding approximate values for the optical constants which are compatible with the values of *A*_cc_ computed from force field parameters. We neglect the zero-frequency term since this is always less than 1*k*_B_*T* in magnitude. Next, we approximate that ν_e_ ≈ 3 × 10^15^ Hz for all beads, such that eq 19 can be numerically inverted to obtain *n*_1_ = *n*_2_ with *n*_3_ = 1 as a function of the Hamaker constant for the chemical interacting with itself in vacuum as calculated above. We assume that ϵ_*i*_ for this bead is a nominal value of 1.3, but note this is effectively negligible compared to that of water. Next, we identify whether the NP material should be treated as metallic or nonmetallic by analyzing the fraction of its constituent atoms which are highly polarizable in terms of their force field parameter ϵ_*i*_ > 12 and quasi-neutral |*q*| < 0.5*e*. If over half of the atoms in the structure meet this definition, the structure is taken to be metallic with a nominal plasma frequency of 5 × 10^15^ Hz and ϵ set to an arbitrarily large number. Otherwise, we extract an effective refractive index through the same procedure as for ABs and again set ϵ_r_ to be a nominal value of 1.3 and assume an electronic adsorption frequency of 3 × 10^15^ Hz. This produces the required set of constants for both the material and AB. Next, we set the medium to be water *n*_3_ = 1.33, ϵ_3_ = 82, ν_w_ = ν_e_, and compute Hamaker constants using the Lifshitz theory based on the extracted approximate optical constants. We stress that this is an approximate procedure and the results are generally only correct to within an order of magnitude and are dependent on the exact force field parametrization used. This is particularly apparent for certain small molecules containing sp3 nitrogen in GAFF parametrizations, which has an unusually large value of ϵ_*i*_ compared to typical atoms, and in general for small molecules containing only one or two heavy atoms due to the low volumes of these beads producing an overestimated numerical density. Given, however, the generally small contribution of the Hamaker potential to UA binding energies, this does not lead to a significant overall error, especially in the context of the limitations of the Hamaker and Lifshitz approach in general.^32^

## Methodology Development and Validation

Here, we summarize the publications introducing or testing the methods described above. The CG scheme to evaluate biomolecule-NP interaction energy, the heatmaps and the ensemble average energy using the united atom one-bead-per-amino acid approach was first introduced in ref (8). This methodology was improved to include the precalculated bead–NP PMFs, including the planar-to-spherical shape correction, in ref (9) and implemented in the UnitedAtom software tool in place of the EspressoMD script previously used. The method to evaluate the Hamaker constant for the interaction between NP materials and AAs using experimental AA radii and refractive indices was introduced in ref (47). This approach was later validated with noble metal NPs. The method has shown a good correlation with experimentally measured adsorption rankings, however, the absolute binding energies were not in agreement with experimental values due to the limitations of the method (“rigid body” model for proteins).^48^ To address the complex variety of available NPs (core–shell NPs, nanocomposites, layered NPs, etc.), the multicomponent “LEGO-like” model was introduced in ref (11) and validated by comparison to experimental results for polymer-coated NPs. The CoronaKMC method for the prediction of protein abundances on solid surfaces was first published in ref (12) and used for the prediction of corona abundances on silica NPs in artificial mixtures of proteins^13^ and for milk proteins on aluminum in refs (14,41).

We have repeated the set of calculations present in ref (13) using the more recent version of this package with results shown in Table 2. Briefly, the NP is taken to be amorphous silica of radius 40 nm and zeta-potential −29 mV. We use AlphaFoldDB structures for all proteins to ensure all residues are present. We have computed relative number abundances for each species *Ñ*_*i*_ to allow a direct comparison between the experimental and simulated results. For the experimental data, we take
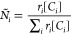
22where *r*_*i*_ is the ratio of the intensity of the gel band for proteins remaining bound to the corona to the intensity of the control gel band, and [*C*_*i*_] is the number concentration of that protein in the medium. We employ this ratio to remove unknown factors such as the dilution of each sample. For the KMC results we simply take the ratio of the number of adsorbed proteins per NP of that type to the total number per NP, *Ñ*_*i*_ = *N*_*i*_/∑_*i*_*N*_*i*_. As can be seen in Table 2, the agreement is generally acceptable other than for the protein BLG (β-lactoglobulin), which is much less represented in the computational corona than in the experimental. We note, however, that the bands associated with this protein are extremely weak in both the NP and control lanes and that BLG is not expected to be present as a monomer at the experimental conditions used,^52^ while the band analyzed corresponds to the molecular weight of the monomer. Since there is no clear band corresponding to a multimer of BLG, we repeated the simulation and analysis with the concentration of the monomer set to an arbitrary small value of 1 × 10^–11^ M under the hypothesis that this protein was not present in large quantities. Doing so produced the results shown in the second section of Table 2, which can be seen to be in much better agreement with the experimental data.

**Table 2:** Relative Abundance of Proteins in the Corona Formed by Silica NPs in an Artificial Mixture of Four Proteins, β-Lacto Globulin (BLG), Lysozyme, Ovalbumin (Ova) and Serotransferrin (sero) as Found by Experiment and Predicted via NPCoronaPredict^a^

protein	uniprot ID	concentration [mg/L]	mum/total (exp)	num/total (KMC)
BLG	P02754	100.0	0.47 ± 0.12	0.25 ± 0.02
Lysozyme	P00698	100.0	0.32 ± 0.07	0.40 ± 0.03
Ova	PP01012	100.0	0.19 ± 0.05	0.25 ± 0.02
Sero	P027878	100.0	0.024 ± 0.006	0.10 ± 0.02
BLG	P02754	2 × 10^–5^	(1.8 ± 0.08) × 10^–6^	0.005 ± 0.004
Lysozyme	P00698	100.0	0.6 ± 0.04	0.52 ± 0.03
Ova	PP01012	100.0	0.35 ± 0.04	0.34 ± 0.03
Sero	P027878	100.0	0.046 ± 0.004	0.13 ± 0.02

a Experimental data was published previously^13^ and here has been postprocessed to express the results in terms of relative abundances, that is, the number of that species adsorbed per NP normalized by the total number adsorbed per NP with errors showing one standard deviation. The errors shown on experimental data are one standard deviation propagated from the uncertainty on the relative band intensities. Errors on KMC data are propagated from approximate standard deviations of  resulting from fluctuations in counts. The lower section indicates a second simulation and analysis performed with the concentration of BLG set to an extremely low value to reflect the near-absence of this protein in the experimental sodium dodecyl sulfate polyacrylamide gel electrophoresis (SDS-PAGE) blot.

As a further validation of the entire package, we have performed a calculation of the corona predicted for silica NPs immersed in human blood to compare to experimental results found previously.^53^ Proteins were selected from the list for blood provided in the Human Protein Atlas,^54,55^ which reports average values for the proteins present in human blood plasma as detected via mass spectroscopy. This resource notes that certain proteins which would otherwise be present at a very high concentration, e.g., human serum albumin, are depleted in this list. Moreover, since these are average values they do not necessarily match the particular concentration range used in the experiment, and so we do not expect perfect agreement. From this list, we selected proteins with a concentration greater than 1 mg/L producing a set of c.a. 280 proteins. The results were matched to UniProtIDs using the online mapping service provided by UniProt,^56^ selecting the IDs corresponding to reviewed genes and limiting the search to *Homo sapiens*. Of these, structures for the majority were successfully automatically retrieved from the AlphaFold Database.^26^ Two of the remaining proteins, P22352 and P49908 were found to contain selenocysteine and so were not predicted by AlphaFold; these were substituted by A0A087X1J7 and A0A182DWH8 respectively due to their high sequence similarity. Six of the remaining proteins did not have full structures but only overlapping fragments provided. For these, the structures for the fragments were fetched and compiled together to produce a single structure for each entire protein using Modeler.^57^ Finally, the genes HBA1 and HBA2 both match to the same structure P69905. We treat these as separate adsorbates during the KMC simulation and average over the values together during postprocessing. Corona simulations for this set of proteins were run for amorphous silica NPs of radius 5, 37, and 501 nm, with the last chosen to test the limit of a planar NP. For all of these a zeta-potential of −10 mV was set, noting that noting that the bulk of the surface charge is already accounted for in the tabulated potentials used. We additionally performed calculations for an Al (110) NP with *R* = 5 nm and surface potential −10 mV to allow for a comparison of the effects of the material. The steady-state corona content was predicted using CoronaKMC operating in standard mode with rate constants rescaled to find the steady state, using spherical models for all but the planar NP, for which we simulate an area of size 80 nm × 80 nm.

**Figure 9:**
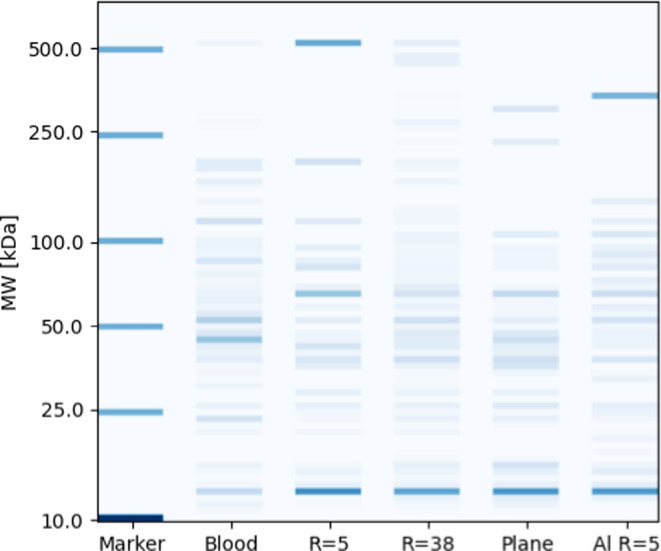
Results from the simulation of the corona formed for NPs in human blood plasma, presented in the style of an SDS-PAGE blot for comparison to experiment. The intensity of each band corresponds to the total mass of protein in that band, normalized within the channel while the location of the band is given by log_10_(MW) as an approximation of where it would appear in a gel experiment.

The results are postprocessed to find the most abundant species in terms of mass per unit area of the NP and the 20 most abundant for the large silica sphere are shown in Table 3. Extended results for all NPs are provided in the Supporting Information. To allow for a quick comparison between the different simulations and what would be observed experimentally, we plot the obtained proteins in the style of an SDS-PAGE blot in Figure 9. It can be immediately seen that increasing the size of the NP favors the adsorption of lighter proteins, which we attribute to the fact that large proteins can pack more efficiently around smaller spheres compared to large spheres or planes and so are more strongly favored for these. The aluminum NP exhibits a different selection of proteins to the silica NP of the same radius, notably including a higher proportion of small proteins, which we attribute to the fact the binding is essentially irreversible for all proteins and so largely reflects the kinetics of adsorption.

**Table 3:** 20 Most Abundant (by Mass) Proteins Predicted in the Corona of a Silica NP of Radius 38 nm Immersed in Blood Plasma, with the Mass Normalized by the Surface Area of the NP^a^

gene name	UniProt ID	description	conc. [mg/L]	num./NP	mass/area [Da/nm^2^]
IGFALS	P35858	IGF-ALS	26.0	4.1	15.7
APOB	P04114	Apo B-100	100.0	0.4	12.0
HSPG2	P98160	HSPG	1.4	0.4	10.9
TNXB	P22105	Tenascin-X	1.5	0.4	10.7
LRG1	P02750	α-2-glycoprotein	42.0	4.2	9.3
LUM	P51884	Lumican	29.0	4.0	8.9
FN1	P02751	Fibronectin	47.0	0.5	7.9
CA1	P00915	Carbonic anhydrase 1	4.6	4.6	7.7
HBA2	P69905	α-globin	14.0	7.9	7.0
HBA1	P69905	α-globin	14.0	7.9	7.0
HBB	P68871	Hemoglobin subunit β	10.0	6.7	6.2
CPB2	Q96IY4	Carboxypeptidase B2	8.5	2.1	5.9
ORM2	P19652	glycoprotein 2	41.0	4.2	5.8
GAPDH	P04406	GAP dehydrogenase	1.4	2.6	5.4
C1RL	Q9NZP8	Complement C1r	15.0	1.7	5.3
PRSS1	P07477	Serine protease 1	100.0	3.3	5.1
CP	P00450	Ceruloplasmin	440.0	0.7	5.0
CPN2	P22792	Carboxypeptidase N-2	25.0	1.4	4.9
HBD	P02042	Hemoglobin subunit delta	6.6	5.1	4.8
SERPINF1	P36955	Pigment factor	44.0	1.8	4.8

a Gene names, the matching UniProt ID and a short description are provided for cross-referencing, as is the concentration of the species in the medium.

A direct comparison of the simulated results to experimental is challenging, given the high dependency of corona measurements on the exact experimental configuration.^58^ Thus, we perform mainly a qualitative assessment here in comparison to the results seen for silica NPs in blood^53^ for which the *R* = 5 nm silica particle is a model for the *d* = 9.6 silica particle of that work, and the *R* = 38 nm is a model for *d* = 76 nm. The results of that work exhibit the same trend with respect to size as observed for the two spherical NPs simulated here, namely, more mass-specific bands for the smaller NP versus a more uniform distribution of bands for the larger NP. For both cases, our results predict a band of increased intensity in the corona compared to blood plasma around 70 kDa which is also observed for both sizes of NP experimentally.

## Applications

Our multiscale model of biomolecular corona formation on solid NPs and surfaces can be generalized to a large variety of systems. It essentially relies only on the existence of an atomistic force field for the target NP material which is compatible with standard force fields for biomolecules, e.g., CHARMM or GAFF. This suggests a further integration with existing methodologies for the prediction of force field parameters via ML techniques is likely to be highly useful in extending the range of materials even further.^59,60^

The model can be used for the prediction of fouling of surfaces in food processing and packaging, screening materials for nanomedicine, toxicology, environmental safety, material design and medical devices. Beyond that, our model benefits from the advancements in computational tools such as PMFPredictor^18^ which makes it possible to predict potentials for arbitrary small molecules of interest such as tannic or humic acid and small metabolites for environmental safety studies or food science. Combined with the fragment-based methodology used in UA, this enables a wide range of biomolecules to be scanned across a variety of NP surfaces.

Our current model treats biomolecules as rigid structures. Incorporating mechanisms to account for protein flexibility, such as generating an ensemble of protein structures rather than using a single structure, can improve the accuracy of the CG models and capture the interactions in diverse environments. Moreover, since one of the outcomes of the modeling is the set of preferred biomolecule orientations on the adsorbent surface, this suggests using these output orientations as starting configurations for more detailed all-atom studies of the corona or individual adsorbed proteins, which can, in turn, be used as improved structures for the inputs to NPCoronaPredict, and this procedure iterated to allow for realistic configurations with a highly optimized runtime.

The NPCoronaPredict pipeline allows for the calculation of numerical descriptors representing the properties of a range of NPs immersed in a biological fluid, but the adsorption energies of the biomolecular fragments and larger proteins are also potentially vital descriptors in categorizing complex structures in a simple numerical form suitable for ML methodologies, e.g., the prediction of further interactions, functionality or safe-by-design development.^61−66^ For these models, it has been demonstrated that simple descriptors obtained from the corona composition can correlate strongly to measures such as NP cell uptake. Here, again, we stress the importance of both the speed and flexibility of our approach to handle essentially arbitrary NP structures. This is vital to be able to provide meaningful descriptors to capture potentially subtle differences in NP structure which may yet lead to significant differences in bioactivity. The scheme we have developed can model crystalline and amorphous, organic and inorganic, modified and pristine NPs on an equal footing, avoiding the risk of requiring extensive categorical or ad-hoc descriptors to account for differences between materials. This is indispensable for fields in which only limited experimental data is available to produce these ML models to limit the potential risk of needing to discard data for materials if these do not fit into an established framework or scan the potential candidate materials even before they are produced.

## Conclusions

We have demonstrated an end-to-end pipeline for the prediction of the corona of adsorbates formed around an NP in a medium containing a mixture of biomolecules and other compounds. Our methodology is sufficiently flexible to allow for corona prediction for a multicomponent NP immersed in media consisting of a large number of varieties of proteins and other adsorbates at a fraction of the computational time which would be required for traditional molecular dynamics simulations. All the code is available open-source for download from ref (22) together with a library of required input which covers a wide range of nanomaterials and biomolecules of interest, and further NP materials or adsorbates can be straightforwardly added by the user as required.

## Data Availability

The NPCoronaPredict package is freely available for download at https://github.com/ucd-softmatterlab. The PMFPredictor software used to produce PMFs and Hamaker constants is available from https://github.com/ijrouse/PMFPredictor-Toolkit. Both packages are provided open source.

## Supporting Information Available

Additional details of UnitedAtom configuration parameters, tables of included scripts, details of included nanomaterials with MD and ML PMFs, schematics of biomolecular fragments (PDF)
Details of file formats, tables of protein corona predictions for four nanoparticles in blood plasma (XLSX)
