# Supplementary material for: NPCoronaPredict: A Computational Pipeline for the Prediction of the Nanoparticle–Biomolecule Corona
Source: J Chem Inf Model. 2024 Sep 26;64(19):7525–43. doi: 10.1021/acs.jcim.4c00434 (PMC11480982; doi:10.1021/acs.jcim.4c00434)
Supplement: Supplementary file 1 — ci4c00434_si_001.pdf [file ci4c00434_si_001.pdf]

Supporting Information

NPCoronaPredict: A computational pipeline for the prediction of the nanoparticle - biomolecule corona

Ian Rouse,* David Power, Julia Subbotina, and Vladimir Lobaskin

University College Dublin

Belfield

Dublin 4

E-mail: ian.rouse@ucd.ie

Simulation Data

In the supplied file Blood_Corona_KMC.xlsx we provide the results from the KMC simulations performed for NPs immersed in a medium representing blood plasma. This file contains four sheets, one per nanoparticle, each containing the following columns:

- Gene: The gene name provided by the Human Protein Atlas.
- UniProtID: The protein ID matched to the gene.
- Entry Name: The UniProt entry name associated with the UniProtID.
- Protein name: The full name associated with the protein, including variants.
- Length: Number of residues present

- Mass: Mass of the protein in Da
- conc_mgL: Concentration in mg/L
- conc_M: Molar concentration
- KMCCount: Number of instances of that protein per simulated NP, averaged over 10 runs and summed over all orientations.
- ShortName: A shortened name used for output.
- Adsorbed Mass [Da/nm^2]: Mass of the protein adsorbed per NP, normalised by the surface area.

UnitedAtom configuration file

In this section, we provide more details on the parameters required for a UnitedAtom configuration file, with an example shown in Figure S1. The most straightforward command-line operation of UnitedAtom is via the supplied RunUA.py script, which automatically generates a configuration file and calls UnitedAtom. This script simplifies the generation of configuration files by allowing the user to chose from predefined materials and automatically filling in the relevant sections of configuration file. The available materials are defined in the files "MaterialSet.csv", corresponding to materials with input potentials found via metadynamics and "MaterialSet-PMFP.csv" for the larger set of materials with potentials obtained via ML with PMFPredictor. The selected material is used to fill in the fields for the pmf-directory, hamaker-file and np-type options to produce a pre-defined material, but this can be edited as needed to allow for custom materials or greater flexibility. If the selected file belongs to MaterialSet.csv then bead parameters are read from beadsets/StandardAABeadSet.csv and used to fill in the corresponding lines in the configuration file, whereas the parameters in pmfp-beadsetdef/PMFP-BeadSet.csv are used otherwise. These latter parameters are more

approximate and automatically generated for a much larger variety of beads based on the summation of forcefield parameters and approximate combination rules.

Table S1 provides an overview of the parameters required for a standard run. All of these must be provided in the configuration file for the program to function including the unused options which are presently disabled but required for backwards compatibility and may be re-enabled in future versions. The exception to this rule is the "enable-[potential]" switches which may be removed or commented out, but in general it is not recommended or necessary to do so. In particular, disabling the surface potential is likely to lead to numerically diverging binding energies since this removes the majority of the repulsive potential at short range. The NP shape parameter is an integer defining whether the adsorption energy should be calculated for spherical (np-shape 1), cylindrical (np-shape 2, 4, 5) or cubic/planar (np-shape 3) co-ordinate systems and further specifies how input PMFs should be shape-corrected. In general, np-shapes 1,2,3 expect PMFs generated for planar surfaces while np-shapes 4,5 require PMFs generated for cylinders of diameter 1.5 nm. Note that UA does not apply error-checking to ensure input PMFs match the expected form and so caution is advised to make sure the selected shape matches the input PMF, especially for low-radius NPs for which these corrections are most significant. For planar surfaces, we recommend the use of np-shape 1 set to a larger radius, $R_{NP} > 100$ nm for reasons of numerical stability in UnitedAtom.

In certain cases, it may be necessary to modify the standard behaviour of UnitedAtom. Table S2 contains some additional parameters which can be used to so. We recommend reading the documentation supplied with UnitedAtom for further details on these. The "recalculate-zp" parameter enables switching UnitedAtom to a mode in which the supplied zeta potentials are interpreted as being defined relative to a reference NP of fixed radius and shape. The electrostatic surface potentials used in the code are then calculated based on the radius and shape of the target NP to produce a constant surface charge density. The disorder-strategy parameter uses the b-factor values supplied in a PDB file to identify

Table S1: Parameters used as input in the configuration file for a standard UnitedAtom run. With the exception of the three switches, all of the above must be supplied.

Parameter	Type	Units	Notes
output-directory	Path	File/Folder	
pdb-target	Path	File/Folder	
nanoparticle-radius	Value/List	nm	
np-type	Integer		see text
pmf-directory	Path	Folder	
hamaker-file	Path	File	
enable-surface	Switch		
enable-core	Switch		
enable-electrostatic	Switch		
simulation-steps	Value		unused
potential-cutoff	Value		unused
potential-size	Value		unused
angle-delta	Value	$\circ$	unused
bjerum-length	Value	nm	unused
debye-length	Value	nm	
temperature	Value	K	
zeta-potential =	Value/List	V	
amino-acids	List	3-letter codes	
amino-acid-charges	List	Elementary charges	
amino-acid-radii	List	nm	

```
#Autogenerated UA Config file
output-directory = CoronaPredictionProjects/C-amorph-3-pmfp-daphnia-10us/results
pdb-target = CoronaPredictionProjects/C-amorph-3-pmfp-daphnia-10us/proteins
nanoparticle-radius = [5.0]
np-type = 1
pmf-directory = surface-pmfp/C-amorph-3-pmfp
hamaker-file = hamaker-pmfp/C-amorph-3-PMFP.dat
enable-surface
enable-core
enable-electrostatic
simulation-steps = 2000
potential-cutoff=5.0
potential-size = 1000
angle-delta = 5.0
bjerum-length=0.716
debye-length=0.785
temperature = 300.0
zeta-potential = [0.0]
amino-acids         = [ ALA, ARG, LYS, HID, HIE, ASP, GLU, SER, THR, ASN, GLN, CYS, G
X09, X0A, X0B, X0C, X0D, X0E, X0F, X0G, X0H, X0I, X0J, X0K, X0L, X0M, X0N, X0O, X0P,
X1H, X1I, X1J, X1K, X1L, X1M, X1N, X1O, X1P, X1Q, X1R, X1S, X1T, X1U, X1V, X1W, X1X,
X2P, X2Q, X2R, X2S, X2T, X2U, X2V, X2W, X2X, X2Y, X2Z, X30, X31, X32, X33, X34, X35,
X3X, X3Y, X3Z, X40, X41, X42, X43, X44, X45, X46, X47, X48, X49, X4A, X4B, X4C, X4D,
amino-acid-charges  = [ 0.0, 1.0, 1.0, 0.0, 0.0, -1.0, -1.0, 0.0, 0.0, 0.0, 0.0, 0.0,
0.0, 0.0, 0.0, 1.0, 0.0, 0.0, 0.0, 0.0, 0.0, 0.0, 0.0, 1.0, 1.0, 1.0, -1.0, 0.0, 0.0,
0.0, 1.0, 1.0, 0.0, 0.0, 0.0, 0.0, 0.0, 0.0, 0.0, 0.0, 0.0, 0.0, 0.0, 1.0, 0.0, -1.0,
0.0, 0.0, 0.0, 0.0, 0.0, 0.0, 0.0, 0.0, 0.0, 0.0, 0.0, 0.0, 0.0, 0.0, 0.0, 0.0, 0.0,
0.0, 0.0, 0.0, 0.0, 0.0, 0.0, 0.0, 0.0, 0.0, 0.0, 0.0, 0.0, 0.0, 0.0, 0.0, 0.0, 0.0,
0.0]
amino-acid-radii    = [ 0.239, 0.365, 0.342, 0.337, 0.337, 0.285, 0.319, 0.244, 0.289
0.321, 0.336, 0.234, 0.364, 0.29, 0.286, 0.255, 0.323, 0.319, 0.327, 0.327, 0.343, 0.
0.321, 0.259, 0.293, 0.37, 0.315, 0.315, 0.289, 0.405, 0.447, 0.409, 0.409, 0.284, 0.
0.334, 0.294, 0.325, 0.153, 0.332, 0.256, 0.259, 0.315, 0.346, 0.333, 0.247, 0.301, 0
0.335, 0.318, 0.343, 0.316, 0.324, 0.344, 0.333, 0.306, 0.305, 0.363, 0.315, 0.328, 0
0.318, 0.338, 0.351, 0.306, 0.333, 0.337, 0.311, 0.326, 0.333, 0.334, 0.302, 0.359, 0
0.158, 0.158, 0.23, 0.411, 0.345, 0.313, 0.315, 0.354, 0.447, 0.352, 0.443, 0.371, 0.
```

Figure S1: A typical configuration file for a single-component NP UnitedAtom run. When executed, this will generate a spherical NP of radius 5 nm using the material parameters for amorphous carbon and compute binding energies for all biomolecules stored in the directory given by pdb-target.

residues which may be disordered and degrade the performance of UA, with different disorder modes treating these residues in different ways. The "enable-fullscan" switch, if included in the configuration file, instructs UA to begin integration of the interaction potential such that the biomolecule's closest approach is computed to allow the NP inside hollow regions of the biomolecule. This ensures that if binding cavities are present the NP can dock into them, but may be physically unrealistic for a small NP and a hollow protein. The "enable-local-boltz" switch results in the mean energies computed in a local cell of ϕ, θ values to be weighted by Boltzmann factors rather than a simple average as is used by default, which typically results in a more favourable binding energy in each local cell. The option "pdb-jitter-magnitude", if set to a value σ greater than zero, performs a slight perturbation of the co-ordinates of

each adsorbate bead (AB) following the generation of a random orientation,

$$x_i \to x_i + \mathcal{N}(0, \sigma) \tag{1}$$

where $\mathcal{N}(\mu, \sigma)$ is a randomly generated, normally distributed variable of mean μ and standard deviation σ. Since this is applied per-axis, the total expected RMSD is equal to $\sqrt{3}\sigma$. For typical PDB resolutions of 0.2 nm this suggests a value of $\sigma \approx 0.1$ nm. Used together with the enable-local-boltz option, this allows a very slight relaxation of the biomolecule in its local environment.

Table S2: Parameters used to modify UnitedAtom behaviour or enable advanced modes. All of these are optional.

Parameter	Type	Units	Notes
omega-angles	List	$\circ$	Final rotation angles
enable-fullscan	Switch		Enables scanning to biomolecule COM
bounding-radius	Value	nm	Override bounding radius for NP
overlap-penalty	Value	$k_B T$	Extra penalty for overlap
recalculate-zp	Integer		See text
calculate-mfpt	Integer		Enable MFPT calculation
disorder-strategy	Integer		See text
disorder-minbound	Float		See text
disorder-maxbound	Float		See text
enable-local-boltz	Switch		See text
pdb-jitter-magnitude	Float	nm	Random AB bead displacement magnitude

CoronaKMC steady-state mode

The default mode for CoronaKMC is to produce a fully time-resolved model of corona evolution. If desorption rates are not too slow compared to adsorption rates and are relatively constant across potential adsorbates, it is possible to fully simulate the corona evolution in a reasonable amount of computational time. We find, however, that in many cases the corona evolution is too slow in this fully dynamic model to reach the final steady state in classic mode, even employing acceleration techniques.[1] We attribute this to the fact that the

rate of acceptance is exponentially suppressed in this mode when the fractional coverage of the nanoparticle is greater than 0.5.[2] Thus, we have added an optional mode in which the simulation attempts to produce the steady-state more rapidly in standard mode. To do so, a pre-processing step is employed in which a scaling factor γ_i is applied to the adsorption rate constant $k_{a,i}$ and desorption rate constant $k_{d,i}$ for each species such that their ratio remains fixed, but the total collision rate $K_i = [C_i]k_{a,i}n_{b,i}$ is equal for all species. In standard mode, this produces the same steady-state adsorption numbers as in the fully-time resolved mode, while accelerating convergence. We note, however, that by doing so the Vroman effect is no longer simulated and the units of time no longer have a physical meaning. Thus, although this rescaling produces the correct end-state, the dynamical evolution is essentially arbitrary. Moreover, even with this rescaling, small adsorbates may block the adsorption of large adsorbates for an extended period of time. Thus, to ensure these have a chance to adsorb and accelerate convergence to the steady-state, displacement mode is automatically enabled for the first 5 time units, and then disabled. The steady-state achieved via displacement mode is typically more dense and contains a higher proportion of strongly-binding adsorbates than standard mode and so it is necessary to allow for an equilibriation period once the simulation returns to classic mode for some large particles to desorb and potentially be replaced by smaller ones. Nonetheless, this is more computationally efficient than the reverse of waiting for a large number of small particles to desorb followed by adsorption of a single large one, which requires a much less probable series of events to take place and so a significantly larger number of simulation steps. The rescaling procedure used here does not produce the correct steady-state for displacement mode if this is left enabled for the entire simulation and so if steady-state mode is enabled then displacement mode will be automatically disabled.

Script library

Here we present a brief overview of the main additional scripts supplied in the NPCoronaPredict repository:

- BuildCoronaCoords.py – Use CoronaKMC output and PDB structures to assemble the final corona for visualisation.
- BuildCoronaParams.py – Convert .uam files to CoronaKMC input.
- ConcatPatches.py – combines input files for CoronaKMC simulations to handle NPs with multiple crystal faces, only recommended if running separate simulations is not possible.
- GetCoronaStats.py – Given CoronaKMC output and biomolecule descriptors, computes corona averages.
- ExtractBindingEnergies.py – Scan input folders for .uam files and record average energies in a tabular format.
- MultiSurfaceAverage.py – Boltzmann average adsorption energies across multiple NP surfaces.
- PreprocessProteins.py – Applies PROPKA and canonical transformation to an input protein.
- tools/ApplyOptimumRotation.py – Given a .uam and .pdb input, rotates the biomolecule to its favoured binding orientation for that .uam file.
- tools/MolToFragments.py – Converts a SMILES code to a set of fragments and provides .pdb output for use in UA.
- tools/plotmap – Generates a heatmap plot for a given .uam file.

Further additional Jupyter and Python scripts are provided for generation of nanoparticles, generation of Hamaker parameters for materials with known optical properties, and visualisation of final results:

1. CalcLifshitzHamaker.ipynb – an example script to demonstrate the generation of files containing Hamaker constants from optical constants, and bead radii from the coordinates obtained from all-atom models together with the atomic Bondi radii.[3,4]

2. VisualiseUAResults.ipynb – a script for post-processing of .uam data files to generate and visualise the lowest energy complexes of proteins adsorbed to the surface of the NP. The script produces the PDB file of the complex for further studies, with the protein represented in all-atom resolution and the NP as the component beads, one "atom" per bead. An example of the produced visualisation for BSA to a pristine silver NP is shown in Figures 4 and 5 of the main text.

3. ViewNP.ipynb – a script for converting .np files into PDB files, followed by their visualisation. The script generates ∗.pdb and ∗.png files for the input ∗.np file describing the structure of the NP, with an example for the core-shell PEGylated AgNP previously employed in Ref.[5] shown in Figure S2.

4. GenerateNanoparticle.py – a set of routines for automated production of core-shell-brush type NPs based on pre-defined bead types and brush densities, including raspberry models for inner cores. This tool is recommended for the bulk generation of NPs when it is not practical to individually generate these using NPDesigner.

5. ApplyOptimumRotation.py – a command line tool for rotation of biomolecules to their optimum binding configuration based on a UA output file, takes command line arguments for batch processing.

We note that further scripts for more specialised or testing purposes are included, but these do not form part of the core repository and may be removed at any time.

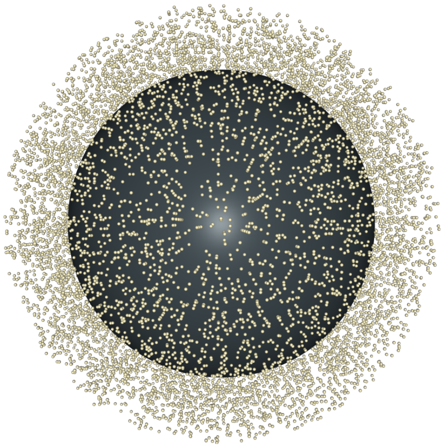

Figure S2: An example of the visualisation for a multi-component core-shell-brush PEGylated silver NP produced by post-processing of an input ∗.np file with the ViewNP.ipynb script, with the dark central bead corresponding to the silver core and the small light-coloured beads representing PEG beads. The initial configuration of the NP was obtained by using GenerateNP.py.

Library of metadynamics PMFs

In Table S3 we present details of the library of PMFs computed using metadynamics techniques and provide information on the sets of fragments and conditions used, with details of the suggested AB parameters in Table S4. The HIS potential is produced via averaging the HID (histidine-delta), HIE (histidine-epislon) and HIP (histidine-protonated) PMFs corresponding to their proportions at physiological conditions. In some cases, potentials for HIP are not available and for these only HIE and HID are included. Note that the PEG-3B set of PMFs represent a small trimer of PEG units rather than a large planar surface and thus must be treated differently to ensure correct behaviour in UnitedAtom. In particular, these require that the LJ-correction option is set to 0 in NPDesigner to prevent UA from applying automatic rescaling and should only be generated as beads of radius 0.2 nm as part of a larger NP. This generally requires an extremely large number of beads and thus we advise the use of the PEG-Slab PMFs to model high-density PEG, reserving the PEG-3B beads for low-density regions. PEG-Slab is computed by post-summation of the metadynamics-derived PEG-3B PMFs over a raspberry model of a planar NP consisting of PEG-3B beads.

Table S3: A summary of the materials included in the metadynamics PMF library as of time of publication. The adsorbate bead section uses the following shorthand to describe available bead sets: F: full AA, S: standard SCA set, G: glycans/carboydrates, X: additional ionised variants, L: lipids and glucose. S- indicates proline is modelled as cyclopropane and glycine is absent.

Material	Force field (nano/bio)	Geometry	Adsorbate beads	Notes	Ref.
Au (100)	INTERFACE/GAFF	Plane	F	No salt	[6]
Au (100/110/111)	INTERFACE FF/CHARMM	Plane	S-LXG		[5]
Ag (100/110/111)	INTERFACE FF/CHARMM	Plane	S-LXG		[7]
Fe (100/110/111)	INTERFACE FF/GAFF	Plane	SL		[8]
Al (100/110/111)	INTERFACE FF/GAFF	Plane	SL		[9]
C-amorph (3× structures)	GAFF/GAFF	Plane	SL		[10]
Graphene (1/2/3 layers)	GAFF/GAFF	Plane	SL		[10]
Graphene oxide	GAFF/GAFF	Plane	SL		[10]
Reduced graphene oxide	GAFF/GAFF	Plane	SL		[10]
CNT	GAFF/GAFF	1.5 nm diameter cylinder	SL		[10]
CNT-OH	GAFF/GAFF	1.5 nm diameter cylinder	SL		[10]
CNT-NH2	GAFF/GAFF	1.5 nm diameter cylinder	SL		[10]
CNT-NH3+	GAFF/GAFF	1.5 nm diameter cylinder	SL		[10]
CNT-COO-	GAFF/GAFF	1.5 nm diameter cylinder	SL		[10]
CNT-COOH	GAFF/GAFF	1.5 nm diameter cylinder	SL		[10]
PEG-3B	INTERFACE FF/CHARMM	Bead	S-LXG		[5]
PEG-Slab	INTERFACE FF/CHARMM	Plane	S-LXG		[5]
Fe2O3 (001)	A.L. /GAFF	Plane	SL		[11]
TiO2 anatase(100, 101)	A. Lyubartsev /GAFF	Plane	SL		[12]
TiO2 rutile (100, 110)	A. Lyubartsev/GAFF	Plane	SL		[12]
SiO2 (quartz, amorphous)	INTERFACE FF/GAFF	Plane	SL		[11]

Table S4: AA radii for Hamaker potentials computed using different methodologies: forcefield parameters and convex hull (FF-CH), zero-crossing of LJ self-interaction (LJ0), weighted average of vdW and maximum radius of gyration (DP/SA), the methodology used in[7] and described in *CalcLifshitzHamaker.ipynb* notebook, and values extracted from solution-phase volumes (S).[13] Note that all radii here are calculated for the full molecule including the AA functional group to better reflect their behaviour in the bulk of a protein.

Chemical	r (FF-CH) [nm]	r (LJ0) [nm]	r (DP/SA) [nm]	r (JS) [nm]	r(S) [nm]
ALA	0.28	0.34	0.32	0.27	0.29
ARG	0.37	0.54	0.43	0.35	-
ASN	0.32	0.35	0.36	0.31	0.31
ASP	0.31	0.35	0.36	0.31	0.30
CYS	0.30	0.32	0.35	0.30	-
GLN	0.34	0.44	0.39	0.32	0.33
GLU	0.33	0.36	0.38	0.32	0.32
GLY	0.25	0.31	0.29	0.27	0.26
HIS	0.34	0.41	0.302	0.33	-
ILE	0.34	0.49	0.40	0.31	0.35
LEU	0.34	0.42	0.40	0.31	0.35
LYS	0.34	0.40	0.41	0.32	0.34
MET	0.34	0.41	0.40	0.33	0.35
PHE	0.36	0.47	0.42	0.34	0.36
PRO	0.28	0.37	0.36	0.29	0.32
SER	0.29	0.31	0.33	0.28	0.29
THR	0.31	0.36	0.36	0.30	0.31
TRP	0.38	0.56	0.45	0.37	0.38
TYR	0.37	0.49	0.43	0.35	0.37
VAL	0.32	0.40	0.38	0.30	0.33

Machine-learning PMFs

In this section, we provide an overview of the full set of small molecules parameterised using PMFPredictor and the associated scripts for use with UnitedAtom. Structures for these molecules are shown in Figure S3 and detailed results (radii, Hamaker constants, SMILES codes) are provided in the NPCoronaPredict repository. Tables S5, S6, S7 and S8 provide details of the available nanomaterials separated into metallic, metal oxide, carbonaceous and miscallenous groups respectively. For all of these, PMFs are generated for the full set of biomolecules and forcefields are either INTERFACEFF or the same as listed in Table S3. All of these materials are defined in the supplied surface-pmfp/MaterialSetPMFP.csv file for importation into NPDesigner, NPCoronaPredict-GUI and RunUA.py.

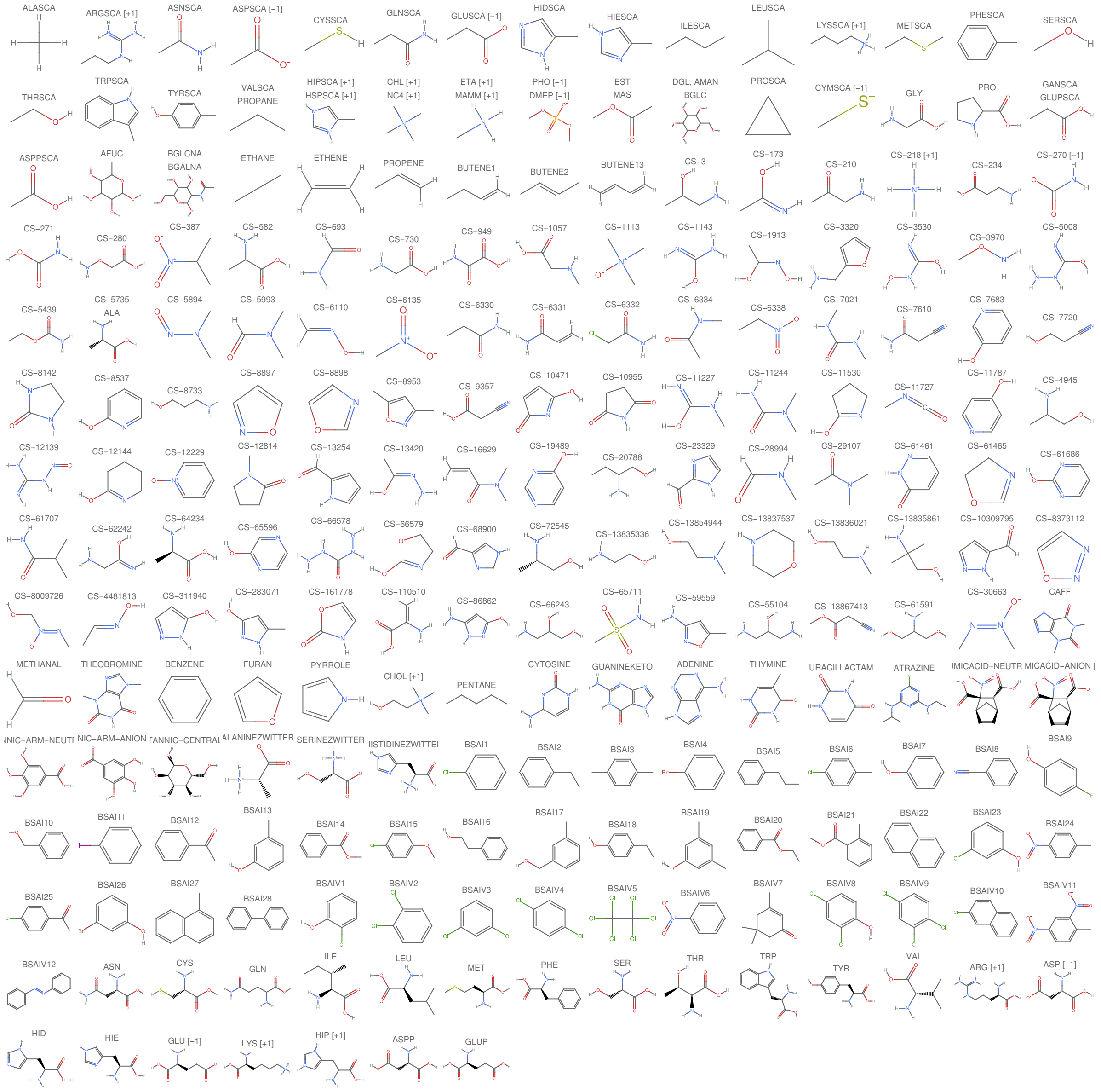

Figure S3: Bead IDs and 2D structures for the full range of small molecules available in the extended bead set. We indicate charges where relevant after the bead ID and structures with identical SMILEs codes are merged but may have multiple bead IDs due to different naming conventions employed by different groups.

Table S5: Table of metallic nanomaterials with PMFs generated via the PMFPredictor method.

Surface ID	Shape	Type	Main elements	Notes
Ag100	plane	Metal	Ag	FCC (100)
Ag110	plane	Metal	Ag	FCC (110)
Ag111	plane	Metal	Ag	FCC (111)
Ag322	plane	Metal	Ag	FCC (322)
Ag332	plane	Metal	Ag	FCC (332)
AlFCC100UCD	plane	Metal	Al	FCC (100)
AlFCC110UCD	plane	Metal	Al	FCC (110)
AlFCC111UCD	plane	Metal	Al	FCC (111)
AuFCC100	plane	Metal	Au	FCC (100)
AuFCC100-Ablate5	plane	Metal	Au	FCC (100) gold, 5% ablation
AuFCC100-Ablate25	plane	Metal	Au	FCC (100) gold, 25% ablation
AuFCC100-Ablate50	plane	Metal	Au	FCC (100) gold, 50% ablation
AuFCC100-Ablate75	plane	Metal	Au	FCC (100) gold, 75% ablation
AuFCC100UCD	plane	Metal	Au	FCC (100)
AuFCC110UCD	plane	Metal	Au	FCC (110)
AuFCC111UCD	plane	Metal	Au	FCC(111)
AuSphere1NM	sphere	Metal	Au	Spherical
Ce-001	plane	Metal	Ce	FCC (001)
Cu001	plane	Metal	Cu	FCC (001)
Cu110	plane	Metal	Cu	FCC (110)
Cu111	plane	Metal	Cu	FCC (111)
Fe001	plane	Metal	Fe	FCC (001)
Fe110	plane	Metal	Fe	FCC (110)
Fe111	plane	Metal	Fe	FCC (111)
Pt001	plane	Metal	Pt	FCC (001)
SS304-111	plane	Metal	Fe, Ni, Cr	Stainless steel, (111)
Au-001-PE	plane	Metal/organic	Au, C	PE brush
Au-001-PEG	plane	Metal/organic	Au, C	PEG brush
GoldBrush	plane	Metal/organic	Au, C	Gold with partial polymer brush

Table S6: Table of metal oxides and sulphides with PMFs generated via the PMFPredictor method.

Surface ID	Shape	Type	Main elements	Notes
Al2O3-001	plane	Metal oxide	Al, O	(001)
CaO001	plane	Metal oxide	Ca, O	(001)
Cr2O3-001	plane	Metal oxide	Cr, O	(001) s
Fe2O3-001O	plane	Metal oxide	Fe, O	(001) hydroxylated
Rutile-100-old	plane	Metal oxide	Ti, O	(100)
SiO2-Amorphous	plane	Metal oxide	Si, O	Amorphous silica
SiO2-Quartz	plane	Metal oxide	Si, O	Quartz
TiO2-ana-100	plane	Metal oxide	Ti, O	Anatase, (100)
TiO2-ana-101	plane	Metal oxide	Ti, O	Anatase, (101)
TiO2-rut-100	plane	Metal oxide	Ti, O	Rutile, (100)
TiO2-rut-110	plane	Metal oxide	Ti, O	Rutile, (110)
ZnO10m10BOND	plane	Metal oxide	Zn, O	Bonded water model, (10-10)
ZnO10m10NB	plane	Metal oxide	Zn, O	Nonbonded water model, (10-10)
ZnO10m10NBDry	plane	Metal oxide	Zn, O	Nonbonded, dehydrated, (10-10)
ZnO1m210BOND	plane	Metal oxide	Zn, O	Bonded water model, (1-210)
ZnO1m210NB	plane	Metal oxide	Zn, O	Nonbonded water model, (1-210)
ZnO1m210NBDry	plane	Metal oxide	Zn, O	Nonbonded, dehydrated, (1-210)
mos2-001	plane	Metal sulphide	Mo, S	(001)
ZnS-NP	sphere	Metal sulphide	Zn, S	Spherical
ZnS-sphalerite-110	plane	Metal sulphide	Zn, S	(110)
ZnS-sphalerite-110-coated	plane	Metal sulphide	Zn, S, C	(110), dense polymer brush

Table S7: Table of carbonaceous nanomaterials with PMFs generated via the PMFPredictor method.

Surface ID	Shape	Type	Main elements	Notes
graphene	plane	Carbonaceous	C	Graphene (one layer)
bi-graphene	plane	Carbonaceous	C	Graphene (two layers)
tri-graphene	plane	Carbonaceous	C	Graphene (three layers)
grapheneoxide	plane	Carbonaceous	C, O	Graphene oxide
redgrapheneoxide	plane	Carbonaceous	C, O	Reduced graphene oxide
C-amorph-1	plane	Carbonaceous	C	Amorphous carbon
C-amorph-2	plane	Carbonaceous	C	Amorphous carbon
C-amorph-3	plane	Carbonaceous	C	Amorphous carbon
CNT15	cylinder	Carbonaceous	C	CNT
CNT15-COO–10	cylinder	Carbonaceous	C, O	CNT, COO- 10%
CNT15-COO–3	cylinder	Carbonaceous	C, O	CNT, COO- 3%
CNT15-COOH-3	cylinder	Carbonaceous	C, O	CNT, COOH 3%
CNT15-COOH-30	cylinder	Carbonaceous	C, O	CNT, COOH 30%
CNT15-NH2-14	cylinder	Carbonaceous	C, N	CNT, NH2 14%
CNT15-NH2-2	cylinder	Carbonaceous	C, N	CNT, NH2 2%
CNT15-NH3+-2	cylinder	Carbonaceous	C, N	CNT, NH3+ 2%
CNT15-NH3+-4	cylinder	Carbonaceous	C, N	CNT, NH3+ 4%
CNT15-OH-14	cylinder	Carbonaceous	C, O	CNT, OH 14%
CNT15-OH-4	cylinder	Carbonaceous	C, O	CNT, OH 4%

Table S8: Table of other nanomaterials with PMFs generated via the PMFPredictor method. All Montmorillionite PMFs correspond to the (001) surface, with this label ommitted for space.

Surface ID	Shape	Type	Main elements	Notes
Gypsum-001	plane	Mineral	Ca, S, O	(001)
Gypsum-010	plane	Mineral	Ca, S, O	(000)
Gypsum-011	plane	Mineral	Ca, S, O	(011)
hydroxyapatite-001-ph5	plane	Mineral	Ca, P, O	(001), pH5
hydroxyapatite-001-ph10	plane	Mineral	Ca, P, O	(001), pH10
hydroxyapatite-001-ph14	plane	Mineral	Ca, P, O	(001), pH14
hydroxyapatite-010-ph5	plane	Mineral	Ca, P, O	(010), pH5
hydroxyapatite-010-ph10	plane	Mineral	Ca, P, O	(010), pH10
hydroxyapatite-010-ph14	plane	Mineral	Ca, P, O	(010), pH14
hydroxyapatite-101-ph5	plane	Mineral	Ca, P, O	(101), pH5
hydroxyapatite-101-ph10	plane	Mineral	Ca, P, O	(101), pH10
hydroxyapatite-101-ph14	plane	Mineral	Ca, P, O	(101), pH14
Kaolinite-001	plane	Mineral	Al, S, O	(001)
LithiumCobaltOxide-001	plane	Mineral	Li, Co, O	(001)
Montmorillonite-001-CEC0	plane	Mineral	Si, Al, Mg, O	(001)
Montmorillonite-001-NaCEC87	plane	Mineral	Si, Al, Mg, O, Na	87mmol/100g Na
Montmorillonite-001-NaCEC90	plane	Mineral	Si, Al, Mg, O, Na	90mmol/100g Na
Montmorillonite-001-NaCEC108	plane	Mineral	Si, Al, Mg, O, Na	108mmol/100g Na
Montmorillonite-001-NaCEC143	plane	Mineral	Si, Al, Mg, O, Na	143mmol/100g Na
Muscovite-001	plane	Mineral	K, Al, Si	(001)
Pyrophyllite-001	plane	Mineral	Al, Si, O	(001)
Tobermorite-004	plane	Mineral	Ca, Si, O	(001)
TricalciumAluminate-Wet-010	plane	Mineral	Ca, Al, O	(004)
TricalciumSilicate-Wet-001	plane	Mineral	Ca, Si, O	(010), hydated
TricalciumSilicate-Wet-010	plane	Mineral	Ca, Si, O	(010), hydated
TricalciumSilicate-Wet-100	plane	Mineral	Ca, Si, O	(100), hydrated
TricalciumSilicate001	plane	Mineral	Ca, Si, O	(001)
TungstenDisulfide-001	plane	Mineral	W, S	(001)
CdSeWurtzite2-10	plane	Semiconductor	Cd, Se	(2-10)

File Formats

In this section we present examples of the formats used for various input files.

- Figure S4: Material definition file
- Figure S5: Bead definition file
- Figure S6: Biomolecule definition file
- Figure S7: NP definition file
- Figure S8: Short-range tabulated potential (PMF) file
- Figure S9: Hamaker constant file
- Figure S10: CoronaKMC input file

```
#Material name, surface folder, Hamaker file, default shape
silicaquartz,surface/SiO2-Quartz,hamaker/SiO2_Quartz.dat,1
silicaamorph,surface/SiO2-Amorphous,hamaker/SiO2_Amorph.dat,1
anatase100,surface/TiO2-ana-100,hamaker/TiO2_Anatase.dat,1
anatase101,surface/TiO2-ana-101,hamaker/TiO2_Anatase.dat,1
rutile110,surface/TiO2-rut-110,hamaker/TiO2_Rutile.dat,1
rutile100,surface/TiO2-rut-100,hamaker/TiO2_Rutile.dat,1
fe2o3,surface/Fe2O3-0010,hamaker/Fe2O3.dat,1
CdSe,surface/CdSe/Wurtzite/2-10/sca,hamaker/CdSe.dat,1
gold,surface/Au/FCC/100/sca,hamaker/Metal.dat,1
carbonblack,surface/C_amorph-1,hamaker/CarbonAmorph.dat,1
cnt,surface/CNT15,hamaker/CNTFilm.dat,4
```

Figure S4: An example of the file defining the types of materials pre-registered with UnitedAtom, defined by a name, surface potential folder, Hamaker file, and the index for the default shape.

Biomolecule files

Biomolecules are defined using PDB formatted files, with the three-letter residue code used to identify the bead type. To allow for rapid coarse-graining of proteins obtained from the PDB repository, only lines starting with "ATOM" are processed and of these, only atoms labelled as "CA" are registered by the program. All other biomolecule files must provide

```
#BeadID,Charge[e],Radius[nm]
ALA,0.0,0.323
ARG,1.0,0.429
ASN,0.0,0.362
ASP,-1.0,0.356
CYS,0.0,0.352
GLN,0.0,0.386
GLU,-1.0,0.376
GLY,0.0,0.285
HIS,0.5,0.302
ILE,0.0,0.401
```

Figure S5: An extract from an adsorbate bead definition file, listing the three-letter code, charge and radius of each adsorbate bead type which can be included in simulations.

```
ATOM    626  CA  SER A  95      61.802 -25.224  25.612  1.00 58.66
ATOM    638  CA  HIE A  97      57.612 -30.371  23.157  0.53 71.64
ATOM   1241  CA  HID A 254      57.612 -30.371  23.157  0.13 71.64
ATOM   1251  CA  HIP A 255      57.612 -30.371  23.157  0.34 71.64
```

Figure S6: A sample input structure defining a biomolecule, extracted from the PDB repository for 1AX8. Here, a histidine residue has been mapped to three ABs reflecting different protonation states with weights stored in the occupation column, while a serine bead is provided with a weight of unity as it has no alternate states. Note that most beads have been omitted for clarity.

beads following this convention and the same formatting with column widths defined as in the standard PDB specification to ensure these are read correctly. The standard AA residue codes e.g. ALA are reserved to describe AAs specifically within the context of a protein, for which UA by default employs the convention that long-range interactions are treated as if it was a full AA, while short-range interactions are computed using only the side-chain analogue (SCA) under the assumption that the amino functional group is fixed within the backbone of the protein and not available for direct binding to the surface of the NP. Two AAs, glycine and proline, require special treatment as they do not have a well-defined SCA. Depending on the source of PMFs, glycine is either omitted or treated as a full AA and proline is either represented as cyclopropane or a full AA. The HIS bead is likewise a special case due to its range of protonation states. In the default bead set it is assigned a charge of

```
#An example NP structure showing the type-bead layout.
#The first line produces a gold core of radius 5nm
TYPE,5.0,0,1,1,1,hamaker/Metal.dat,surface/Au/FCC/100/sca,1.2,1
#These lines produce a shell of thickness 0.5 nm by subtracting
# a 5.5nm bead from a 6nm bead.
TYPE,5.5,0,-1,-1,1,hamaker/TiO2_Anatase.dat,surface/TiO2-ana-101,1.0,1
TYPE,6.0,0,1,1,1,hamaker/TiO2_Anatase.dat,surface/TiO2-ana-101,1.0,1
#Provide co-ordinates for beads of the above types.
BEAD,0,0,0,0
BEAD,1,0,0,0
BEAD,2,0,0,0
```

Figure S7: A sample NP definition file to produce a gold core (bead type 0) with a thin layer of anatase, generated by subtraction of a bead of radius 5.5 nm from a bead of radius 6 nm.

```
# Ag110_ALASCA-AC
# h[nm],U(h)[kJ/mol]
0.20000000000000011,84.206757354268503946
0.20100000000000012,80.171277651549075927
0.20200000000000013,77.750456613161659902
0.20300000000000014,76.020565564960605798
0.20400000000000015,74.484777483726560376
0.20500000000000016,72.908528362895978603
0.20600000000000016,71.209969241495542747
```

Figure S8: An example of the input required to describe the short-range surface potential for a particular adsorbate bead (here an alanine side chain analogue) to a given NP type (silver, 110 surface plane).

0.5e and the PMFs are typically supplied for delta (HID), epsilon (HIE) and fully protonated (HIP) configurations, one of which must be used for the HIS bead unless pre-processing is performed as discussed later. Other molecules may be represented using any three-letter tag, and a list of suggested assignments is included in the repository for the extended beat set covering a range of common biomolecular fragments. For example, in this set the bead code "X1B" corresponds to a CH4 molecule (alanine side-chain) for both PMF and Hamaker constant, while "X6H" produces full-AA alanine for both PMF and Hamaker constant. The occupation field is used to scale the total potential for that particular bead, which may be used to represent disordered residues or molecules that may potentially exhibit multiple charge states at a given pH. The b-factor column is used to identify beads which do not occupy a well-defined position in the biomolecule which can be optionally disabled entirely or moved to the centre of the molecule, depending on options set in the UA configuration

#Name	kT	Joules	kJ/mol
ALA	4.442	1.840E-20	11.105
ARG	5.182	2.146E-20	12.954
ASN	5.519	2.286E-20	13.797
ASP	5.630	2.332E-20	14.076
CYS	5.444	2.255E-20	13.611
GLN	5.257	2.177E-20	13.143
GLU	5.068	2.099E-20	12.671
GLY	5.444	2.255E-20	13.611
HIS	5.630	2.332E-20	14.076
ILE	3.946	1.635E-20	9.866

Figure S9: An extract from a file used to parameterise the long-range NP-AB potentials, listing Hamaker constants for a range of biomolecular fragments to SiO_2 quartz across an aqueous medium.

```
AF-A0A162CU06-F1-model_v4-P1:2.5-2.5 9.928012172604746e-11 1.5754167388639813 149386407.61609712 122369638.61136691 -0.19949 4.575150993960135
AF-A0A162CU06-F1-model_v4-P1:2.5-7.5 9.928012172604746e-11 1.578620442618941 149697140.5647671 120962936.66722803 -0.21313 4.58951744533828 3
AF-A0A162CU06-F1-model_v4-P1:2.5-12.5 9.928012172604746e-11 1.587024164622228 150512391.59243006 120043648.83806957 -0.22619 4.6272476236911855
AF-A0A162CU06-F1-model_v4-P1:2.5-17.5 9.928012172604746e-11 1.6001541318399464 151786600.40726504 119197953.95817159 -0.24169 4.686326623086239
AF-A0A162CU06-F1-model_v4-P1:2.5-22.5 9.928012172604746e-11 1.6164287526460799 153366766.8947245 118715778.6997152 -0.2561 4.759770434593677
AF-A0A162CU06-F1-model_v4-P1:2.5-27.5 9.928012172604746e-11 1.6482225950756026 156456258.6965198 118908759.69398579 -0.27442 4.903917536474036
AF-A0A162CU06-F1-model_v4-P1:2.5-32.5 9.928012172604746e-11 1.6708068526181514 158652847.63208964 118274532.2821289 -0.29371 5.006829993397862
AF-A0A162CU06-F1-model_v4-P1:2.5-37.5 9.928012172604746e-11 1.6944449771502295 160953736.86905175 116941870.1550981 -0.31944 5.114990606377687
AF-A0A162CU06-F1-model_v4-P1:2.5-42.5 9.928012172604746e-11 1.7180905086125988 163257192.22062528 115162805.09265997 -0.34898 5.22362652915894
AF-A0A162CU06-F1-model_v4-P1:2.5-47.5 9.928012172604746e-11 1.7407062569439022 165462060.3767916 114998826.74579735 -0.36382 5.3279307622895375
AF-A0A162CU06-F1-model_v4-P1:2.5-52.5 9.928012172604746e-11 1.7612777684331722 167469102.6258533 111527240.70478562 -0.40653 5.423135411182244
AF-A0A162CU06-F1-model_v4-P1:2.5-57.5 9.928012172604746e-11 1.7788166345935363 169181376.69422254 113321779.06237996 -0.40074 5.504544943565923
AF-A0A162CU06-F1-model_v4-P1:2.5-62.5 9.928012172604746e-11 1.797235682882803 170980681.42637601 103805668.18220186 -0.49903 5.590271199439046
```

Figure S10: An extract from an input file for the CoronaKMC script. The first entry gives a unique identifier for the adsorbate, including its orientation, followed by a concentration in the medium, effective radius, adsorption and desorption rate constants, adsorption affinity, and occupied area.

file. The remaining PDB fields are presently unused.

Optional pre-processing of proteins by PreprocessProteins.py consists of two stages. Firstly, the PROPKA software tool (if available) is run to compute the pKa of residues which may exist in multiple charge states. The target pH of the solution is then employed to predict the probability for the residue to exist in each of these states, and the residue is replaced with multiple "fractional beads" according to this weight. For example, a histidine residue (HIS) is replaced by three beads corresponding to the epsilon-histidine (HIE), delta-histidine (HID), and protonated histidine (HIP) beads to avoid the requirement to manually select a suitable HIS potential, see Fig. S6 for an example. Secondly, a principal axes transformation is performed to rotate the protein into "canonical form", in which the z-axis is associated with the smallest moment of inertia and the y-axis with the second smallest, and

further rotations are performed to ensure that the projection of the electric dipole moment onto these two axes is positive. The goal of this set of rotations is to provide a well-defined initial state for the protein such that later rotations are defined with respect to this rather than the potentially random orientation obtained from the initial source.

References

(1) Dybeck, E. C.; Plaisance, C. P.; Neurock, M. Generalized temporal acceleration scheme for kinetic monte carlo simulations of surface catalytic processes by scaling the rates of fast reactions. *J. Chem. Theory Comput.* **2017**, *13*, 1525–1538.

(2) Rouse, I.; Lobaskin, V. A hard-sphere model of protein corona formation on spherical and cylindrical nanoparticles. *Biophys. J.* **2021**, *120*, 4457–4471.

(3) Bondi, A. v. van der Waals volumes and radii. *J. Phys. Chem* **1964**, *68*, 441–451.

(4) Zhao, Y. H.; Abraham, M. H.; Zissimos, A. M. Fast calculation of van der Waals volume as a sum of atomic and bond contributions and its application to drug compounds. *J. Org. Chem.* **2003**, *68*, 7368–7373.

(5) Subbotina, J.; Rouse, I.; Lobaskin, V. In silico prediction of protein binding affinities onto core–shell PEGylated noble metal nanoparticles for rational design of drug nanocarriers. *Nanoscale* **2023**, *15*, 13371–13383.

(6) Power, D.; Rouse, I.; Poggio, S.; Brandt, E.; Lopez, H.; Lyubartsev, A.; Lobaskin, V. A multiscale model of protein adsorption on a nanoparticle surface. *Model. Simul. Mater. Sci. Eng* **2019**, *27*, 084003.

(7) Subbotina, J.; Lobaskin, V. Multiscale Modeling of Bio-Nano Interactions of Zero-Valent Silver Nanoparticles. *J. Phys. Chem. B* **2022**, *126*, 1301–1314.

(8) Mosaddeghi Amini, P.; Subbotina, J.; Lobaskin, V. Milk Protein Adsorption on Metallic Iron Surfaces. *Nanomaterials* **2023**, *13*, 1857.

(9) Amini, P. M.; Rouse, I.; Subbotina, J.; Lobaskin, V. Multiscale modelling of biomolecular corona formation on metallic surfaces. *Beilstein J. Nanotechnol.* **2024**, *15*, 215–229.

(10) Saeedimasine, M.; Brandt, E. G.; Lyubartsev, A. P. Atomistic perspective on biomolecular adsorption on functionalized carbon nanomaterials under ambient conditions. *J. Phys. Chem. B* **2020**, *125*, 416–430.

(11) Lyubartsev, A.; Brandt, E.; Saeedimasine, M. Adsorption free energies and potentials of mean- force for interactions between amino acids, lipid fragments, and nanoparticles. 2020; https://doi.org/10.5281/zenodo.4314912.

(12) Rouse, I.; Power, D.; Brandt, E. G.; Schneemilch, M.; Kotsis, K.; Quirke, N.; Lyubartsev, A. P.; Lobaskin, V. First principles characterisation of bio–nano interface. *Phys. Chem. Chem. Phys.* **2021**, *23*, 13473–13482.

(13) Counterman, A. E.; Clemmer, D. E. Volumes of individual amino acid residues in gas-phase peptide ions. *J. Am. Chem. Soc.* **1999**, *121*, 4031–4039.
